# *Cordyceps militaris* improves the survival of Dahl salt-sensitive hypertensive rats possibly via influences of mitochondria and autophagy functions

**DOI:** 10.1016/j.heliyon.2017.e00462

**Published:** 2017-11-24

**Authors:** Kentaro Takakura, Shogo Ito, Junya Sonoda, Koji Tabata, Motoko Shiozaki, Kaoru Nagai, Masahiro Shibata, Masato Koike, Yasuo Uchiyama, Takahiro Gotow

**Affiliations:** a Laboratory of Cell Biology, College of Nutrition, Koshien University, Takarazuka, Hyogo 665-0006, Japan; b Department of Cardiovascular Surgery, Osaka University Graduate School of Medicine, Suita, Osaka 565-0871, Japan; c Laboratory of Cellular Biochemistry, College of Nutrition, Koshien University, Takarazuka, Hyogo 665-0006, Japan; d Department of Morphological Science, Kagoshima University Graduate School of Medical and Dental Sciences, Kagoshima 890-8580, Japan; e Department of Cell Biology and Neuroscience, Juntendo University School of Medicine, Tokyo 113-8421, Japan

**Keywords:** Neuroscience, Biochemistry, Physiology, Cell biology, Toxicology

## Abstract

The genus *Cordyceps* and its specific ingredient, cordycepin, have attracted much attention for multiple health benefits and expectations for lifespan extension. We analyzed whether *Cordyceps militaris* (CM), which contains large amounts of cordycepin, can extend the survival of Dahl salt-sensitive rats, whose survival was reduced to ∼3 months via a high-salt diet. The survival of these life-shortened rats was extended significantly when supplemented with CM, possibly due to a minimization of the effects of stroke. Next, we analyzed the effect of CM on hypertension-sensitive organs, the central nervous systems (CNS), heart, kidney and liver of these rats. We attempted to ascertain how the organs were improved by CM, and we paid particular attention to mitochondria and autophagy functions. The following results were from CM-treated rats in comparison with control rats. Microscopically, CNS neurons, cardiomyocytes, glomerular podocytes, renal epithelial cells, and hepatocytes all were improved. However, immunoblot and immunohistochemical analysis showed that the expressions of mitochondria-related proteins, ATP synthase β subunit, SIRT3 and SOD2, and autophagy-related proteins, LC3-II/LC3-I ratio and cathepsin D all were reduced significantly in the CNS neurons, but increased significantly in the cells of the other three organs, although p62 was decreased in its expression in all the organs tested. Activity of Akt and mTOR was enhanced but that of AMPK was reduced in the CNS, while such kinase activity was completely the opposite in the other organs. Together, the influence of CM may differ between mitochondria and autophagy functioned between the two organ groups, as mitochondria and autophagy seemed to be repressed and promoted, respectively, in the CNS, while both mitochondria and autophagy were activated in the others. This could possibly be related to the steady or improved cellular activity in both the organs, which might result in the life extension of these rats.

## 1 Introduction

The *Cordyceps* species has been utilized for medicinal purposes and is believed to be effective against age-related diseases including cancers/tumors (Yang et al., 2012), diabetes/renal injury (Liu et al., 2016), and neurodegeneration (Hwang et al., 2008). Cordycepin, a 3’deoxyadenosine, is a specific and active ingredient of the *Cordyceps* species. This compound is a derivative of the nucleoside adenosine, and it terminates chain elongation during mRNA translation with the absence of oxygen at the 3′ position of ribose (Wang et al., 2014), and, therefore, we speculated that *Cordyceps* would suppress cell division/proliferation by inhibiting mRNA and protein synthesis, which would result in an effective repression of cancer/tumor cells. In addition to killing cancer or tumor cells (Wang et al., 2014), cordycepin has various beneficial effects against oxidative stress (Wang et al., 2015), obesity-related disorders (Takahashi et al., 2012), diabetes (Ma et al., 2015) and neurodegeneration (Jin et al., 2014), as shown by the effect of *Cordyceps.*

*Cordyceps militaris* (CM) is known to contain much more cordycepin than *Cordyceps sinensis* that is traditionally and widely used for experiments as well as in the treatment of many diseases (Xu et al., 2016). Since cordycepin is a compound produced specifically from *Cordyceps*, we postulated that the unique beneficial/protective effects of anti-aging brought about by *Cordyceps*, particularly those by CM, could be due to the presence of this compound. However, the anti-aging effects and lifespan extension by *Cordyceps sinensis* is expected, because this has already been demonstrated on a fly, *Drosophila melanogaster* (Zou et al., 2015). Further, another ingredient of CM, polysaccharide, was considered to be an antiaging factor in a study involving a D-galactose-induced aging mouse model (Li et al., 2010), but this chemical is also found in other fungi or mushrooms. In this study, we used CM, that provided much cordycepin, in order to elucidate how CM influences the lifespan of a mammal.

To analyze the lifespan of the rat, we used a Dahl salt-sensitive rat model, because the lifespan of this rat can be reduced to ∼3 months when treated with a high-salt (8% NaCl) diet, possibly by causing hypertension-induced diastolic heart failure or stroke. Eisenberg et al. (2016) delayed the progression of diastolic heart failure in high-salt treated Dahl rats by reducing systemic blood pressure, although the lifespan was not analyzed. In that study, titin phosphorylation was enhanced and cardiac hypertrophy was prevented in these Dahl rats by treatment with a polyamine, spermidine, that was shown to extend the lifespan by activating the autophagic function in yeast, worm and fly (Eisenberg et al., 2009; Morselli et al., 2011). The life-shortened Dahl rats created via a high-salt diet were suitable for repeated analysis that required only a short experimental term, which allowed for a more distinct decision as to whether CM really prolongs lifespans.

If the life-shortened Dahl rats recover or their survival is prolonged with CM treatment, we next analyze the cellular level of several organs that are susceptible to hypertension, such as the central nervous system (CNS), cardiovascular system, kidney and liver. The aim of the current study is to determine how cells are improved morphologically as well as biochemically by focusing on possible cell survival pathways (Park et al., 2014). No report could be located in the literature concerning the influence of CM as it was related to the mitochondria and/or autophagy in various cells of normal organs. The prediction is that if CM supplementation really prolongs the survival of Dahl rats, then CM must exert significant effect on mitochondria or autophagy function related to lifespan extension (Madeo et al., 2015; Sanz, 2016).

## 2 Materials and methods

### 2.1 Animals

The ARRIVE Guidelines Checklist are shown in Supplementary Material (Table S1). Dahl salt-sensitive rats (DIS/Eis), 4 weeks of age, were obtained from SHIMIZU Laboratory Supplies Co., Ltd. (Kyoto, Japan). Animal experimental protocols used for the current study were approved by the Animal Care and Ethical Use Committee of Koshien University (approved number: 2601), in accordance with the Guide for the Care and Use of Laboratory Animals of Koshien University and the National Institute of Health. The Dahl rats were maintained on a standard diet (MF, Oriental Yeast Co. Ltd., Tokyo, Japan) for 1 week in our animal room. At 5 weeks of age, the rats were randomly divided into two groups. One, which was used as the control group, was fed a high-salt diet containing 8% NaCl, and the other, called the *Cordyceps militaris* (CM)-treated group, was fed a high-salt diet plus 3% CM powder. The CM powder was generously supplied by the JAPAN SILK BIO R&D CENTER (Kobe, Japan). Since the concentration of the NaCl was slightly reduced in the CM-treated group due to the addition of 3% CM, the standard high-salt diet for the control group was made equivalent to that of the CM group in terms of NaCl concentration. These diets were maintained for various experiments, or until death, in order to analyze the effect on survival. For morphological (light and electron microscopy), immunohistochemical and immunoblot analyses, Dahl rats 10–12 weeks of age were used, and basically similar results were recorded in many of these stages for both the control and CM-treated groups, in which the strokes were caused. Thus, the morphological and biochemical data shown here were all from 11 weeks of age, which allowed us to constantly carried out the experiments using age-matched animals from both control and CM-treated groups, because stroke tended to not occur at 10 weeks of age, and, instead, control rats tended to die abruptly at 12 weeks of age. Four animals were used for light/electron microscopy, 6 for immunohistochemistry and 5 for immunoblot from both groups. In addition, 1 ∼ 2 animals 10 or 12 weeks of age were preliminary analyzed in both groups. The animals were singly caged in specific pathogen-free conditions and allowed ad libitum access to food and water, with a constant temperature of 23 °C, at 55% humidity with controlled lighting (12 hr light-dark cycle). Animal survival was assessed using the log-rank test in order to compare the differences in the Kaplan-Meier survival curves between control and CM-treated rats. The body weights of the rats and the amounts of food and water they ate and drink daily, respectively, were measured weekly. Blood pressure was measured via the tail-cuff method (BP98-RCF, Softron Co. Ltd., Tokyo, Japan) after individual rats were wrapped with a Softron Rat-Pocket-O (BP98-PKO) with temperature adjusted to 37.5 °C. These obtained values, shown as the mean ± SEM, were compared using one-way ANOVA, followed by a Bonferroni’s post hoc test in control and CM-treated mice (Shiozaki et al., 2011). *P* values < 0.05 were considered statistically significant.

### 2.2 Antibodies

Antibodies, used for immunoblot and/or immunohistochemistry, are shown in Table 1.

**Table 1:** Primary and secondary antibodies used in the current experiments.

Primary antibody	Application	Source/Country
anti-LC3B (rabbit polyclonal)	immunoblot	ab48394, Abcam/Japan
	immunohistochemistry	
anti-cathepsin D (rabbit polyclonal)	immunoblot	ab826, Abcam/Japan
	immunohistochemistry	
anti-p62 (SQSTM1) (rabbit polyclonal)	immunoblot	PM045, MBL/Japan
anti-SIRT1 (H-300) (rabbit polyclonal)	immunoblot	sc-15404, SantaCruz /USA
anti-SIRT3 (rabbit polyclonal)	immunoblot	ab75434, Abcam/Japan
anti-ATPB antibody (3D5) (mouse monoclonal) (mitochondrial ATP synthase β subunit protein)	immunoblot	ab14730, Abcam/Japan
	immunohistochemistry	
anti-SOD2/MnSOD (rabbit polyclonal)	immunoblot	ab13534, Abcam/Japan
	immunohistochemistry	
anti-Akt(pan) (C67E7) (rabbit polyclonal)	Immunoblot	4691, Cell Signaling Technology/Japan
anti-phospho-Akt (Ser473) (D9E) (rabbit polyclonal)	immunoblot	4060, Cell Signaling Technology/Japan
anti-AMPKα1 + AMPKα2 (rabbit polyclonal)	immunoblot	ab39644, Abcam/Japan
anti-phospho-AMPKα (Thr172) (40H9) (rabbit monoclonal)	immunoblot	2535, Cell Signaling Technology/Japan
anti-mTOR (rabbit polyclonal)	immunoblot	2972, Cell Signaling Technology/Japan
anti-phospho-mTOR (Ser2448) (D9C2) (rabbit monoclonal)	immunoblot	5536, Cell Signaling Technology/Japan
anti-synaptophysin1 (rabbit polyclonal)	Immunoblot Immunohistochemistry	101002, Synaptic Systems/Germany
anti-neurofilament H (NF-H), Nonphosphorylated (SMI32) (mouse monoclonal)	immunoblot	801701, BioLegend/USA
	immunohistochemistry	
anti-MAP2A, 2B, 2C (mouse monoclonal)	immunoblot	MAB364, Chemicon/USA
	immunohistochemistry	
anti-desmin (mouse monoclonal)	immunoblot immunohistochemistry	M0760, Dako/Japan
anti-GAPDH (rabbit polyclonal)	immunoblot	NB100-56875, Novus Biologicals/USA

**Secondary antibody**		

anti-mouse immunoglobulins (goat polyclonal)	immunoblot	P0477, Dako/Japan
anti-rabbit immunoglobulins (swine polyclonal)	immunoblot	P0399, Dako/Japan
anti-mouse IgG(H + L) AlexaFluor 488 (goat polyclonal)	immunohistochemistry	A-11001, ThermoFisher/Japan
anti-rabbit IgG(H + L) AlexaFluor 488 (goa polyclonal)	immunohistochemistry	A-11008, ThermoFisher/Japan
anti-mouse IgG(H + L) AlexaFluor 555 (goat polyclonal)	immunohistochemistry	A-21422, ThermoFisher/Japan
anti-rabbit IgG(H + L) AlexaFluor 555 (goat polyclonal)	immunohistochemistry	A-21428, ThermoFisher/Japan

### 2.3 Light and electron microscopy

Dahl rats were anesthetized by intraperitoneal injection of chloral hydrate (0.35/Kg body weight) and fixed by intracardiac perfusion with 1% glutaraldehyde and 1% paraformaldehyde in 0.1 M phosphate buffer, pH 7.4. After rinsing with phosphate buffered saline (PBS), pH 7.4, slices of fixed tissues, obtained from brain, spinal cord, heart, kidney and liver, were postfixed with 1% OsO_4_ for 4 hr at 4 °C, treated with 1% uranyl acetate for 1 hr at 4 °C, dehydrated in ethanols, and embedded in Epon812 (TAAB Laboratories Equipment Ltd, Berks, UK). For light microscopy, 1 μm thick sections were cut and stained with toluidine blue. Photographs of various tissues were taken using a digital microscope (COOL SCOPE, Nikon Instruments, Inc., Tokyo, Japan). For electron microscopy, thin sections, stained with uranyl acetate and lead citrate, were examined with a Hitachi H-300 or H-7100 electron microscope. Electron micrographs were produced with a film scanner (GT-9800F, Epson, Tokyo, Japan), and both light and electron microscopic images were compiled using Adobe Photoshop Element software.

### 2.4 Quantification of the distribution density of mitochondria and neuronal cytoplasmic density on electron micrographs

The distribution density of mitochondria in the perikaryal cytoplasm of cortical neurons, hippocampal pyramidal cells, Purkinje cells, and anterior horn cells was analyzed using Image J software. The number of mitochondria in cardiomyocytes was directly counted along myofibrils on micrographs. Cytoplasmic densities of cortical neuronal cell bodies, hippocampal pyramidal cell bodies and dendrites, Purkinje cell bodies and dendrites, anterior horn cell bodies, and myelinated axons in the anterior horn were analyzed using WinROOF software (Ver.6.1.1) (Mitani Corp., Tokyo, Japan). The values were obtained from more than 20 cells in the respective neurons and cardiomyocytes from four control and CM-treated rats, expressed as the mean ± SD, and these were compared via a Student’s t test. *P* < 0.05 was considered statistically significant.

### 2.5 Immunohistochemistry

The Dahl rats anesthetized with chloral hydrate were perfused through the ascending aorta with PBS, which was followed with 4% paraformaldehyde in 0.1 M phosphate buffer. Respective organs, the same as those analyzed for light/electron microscopy, were removed, rinsed with PBS, cut and immersed in 30% sucrose in PBS, pH 7.4. They were embedded in an O.C.T. compound (Sakura Finetek USA), frozen in dry ice powder, and 7 μm frozen tissue sections were then cut with a cryostat (Leica CM1510, Germany). The sections were mounted on MAS-coated glass slides (Matunami Gkass Ind., Ltd., Osaka, Japan), and were treated with PBS containing 1% bovine serum albumin (Wako Pure Chemical Industries, Osaka, Japan) and 0.05–2.0% Tween 20. They were then incubated with each of the primary antibodies, diluted in 1–20 μg protein/ml, and with the appropriate fluorescence-conjugated secondary antibody. After rinsing with PBS, they were mounted with an anti-fading medium, ProLong (ThermoFisher), and were examined using a confocal laser scanning microscope (LSM 5 PASCAL, Carl Zeiss Co. Ltd., Germany).

### 2.6 Fluorescent staining for lipid droplets in liver tissue

Frozen sections of liver tissues, 7 μm in thickness, similar to those used for the immunohistochemistry, were mounted on the glass slides, as mentioned above. After the sections were rinsed with PBS, they were treated with fluorescent dye BODIPY 493/503 (32412w, Invitrogen). They were rinsed with PBS again, mounted with anti-fading medium, ProLong, and were examined using a confocal laser scanning microscope (Shiozaki et al., 2011).

### 2.7 Quantification of fluorescent intensities in immunolabeled and lipid-stained images

Laser microscopic images were transferred to a personal computer (Fujitsu Siemens, Scenic L i850, Tokyo, Japan) using LSM 5 PASCAL Software Ver. 2.8 and were processed using Adobe Photoshop Element (Adobe Systems, San Jose, CA, USA). In respective immunolabeled samples, more than 10 images, edited using Adobe Photoshop Element, from six control and CM-treated Dahl rats, were quantified using WinROOF software (Ver.6.1.1). The data were expressed as the mean ± SD, and were then compared statistically using a Student’s t-test (Shiozaki et al., 2011; Hayakawa et al., 2013). *P* < 0.05 was considered statistically significant.

### 2.8 Immunoblotting

The Dahl rats were anesthetized with chloral hydrate and decapitated, and the same organs as those used for morphological/immunohistochemical analyses, were removed and cut into small pieces. These tissue pieces were homogenized with a PTFE-glass homogenizer in 0.32 (for CNS) or 0.25 M (for other organs) sucrose buffer (3 mM Tris-HCl, pH 7.2, 0.1 mM EGTA) containing a protease inhibitor cocktail (Roche Applied Science, Germany) at 4 °C. They were then centrifuged at 1,000 g for 10 min at 4 °C to remove the nuclear fraction. The supernatant was measured for protein concentration using a BCA assay kit (Pierce Chemical Co., IL). Then, 10–20 μg of protein was subjected to SDS-PAGE with 5, 7.5 or 12.5% gels, and was then transferred to PVDF membranes (GE Healthcare) using the Mini-transblot system (170–3930, Bio-Rad, Lab., CA). Protein blots in PVDF membranes were treated with 5% skim milk in Tris-buffered saline (TBS) and incubated with each of the primary antibodies (0.1–1 μg protein/ml) for 1–2 h either at room temperature or overnight at 4 °C. After washing with TBS, the membranes were incubated with the appropriate HRP-labeled secondary antibody for 1 h, and immunoreactive signals were then visualized using the ECL system (GE Healthcare) (Shiozaki et al., 2011; Hayakawa et al., 2013).

### 2.9 Quantifying the intensity of immunoblotted protein bands

The protein bands generated from each antibody of the respective control and CM-treated rat tissues were used for quantification. Respective protein bands on the film (Hyperfilm ECL, GE Healthcare) from the organs mentioned above were scanned and analyzed using WinROOF software (Ver.6.1.1). We used the same size rectangle box to surround each band, and analyzed the intensity of each band using the WinROOF program. Ten or more samples from 5 different animals in control and CM-treated groups were selected. Data, indicated as the mean ± SD, as mentioned in the quantification of fluorescent intensity, were compared statistically using a Student’s t-test. *P* < 0.05 was considered statistically significant.

## 3 Results

### 3.1 Changes in body weight, food and water intake, blood pressure and survival between control and CM-treated Dahl rats

First, we determined the daily amount of CM that should be administered to the Dahl rats. Since a high dose of CM (3 g/kg (body weight)/day) was considered harmful to the kidneys of rats (Zhou and Yao, 2013), we selected an amount that would approximate such a dose to more clearly define the influence of CM and either the benefit or harm to these hypertensive rats that had been fed a high-salt diet. The 3 g of CM/kg/day approximated the 3% of CM used in the high-salt diet, which was the maximum diet for Dahl rats that were ∼ 250 g in weight at around 7–9 weeks of age and fed ∼ 25 g of the diet/day. At this age, the Dahl rats consumed ∼ 0.75 g CM daily, which was equivalent to the amount of CM (3 g/kg/day) reported by Zhou and Yao (2013).

**Fig. 1:**
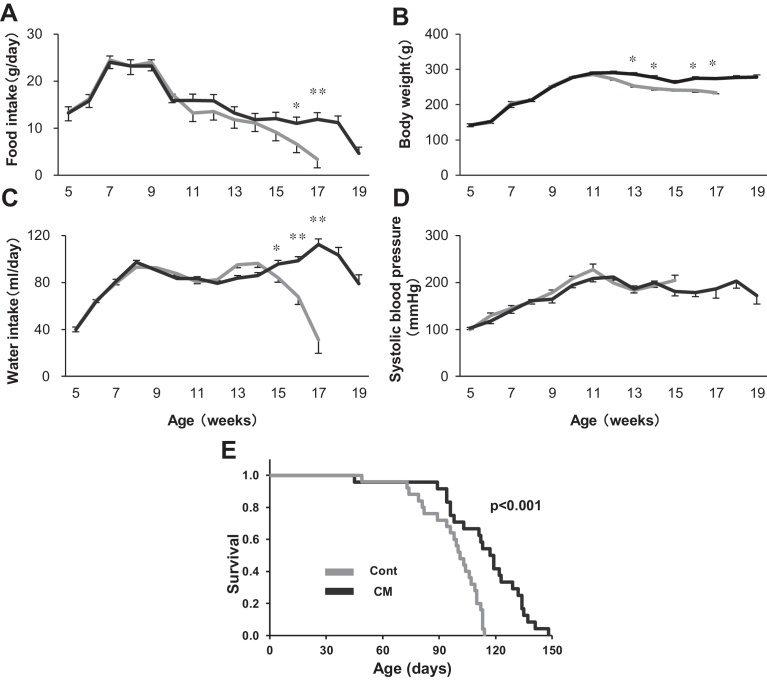
Food (A) and water (B) intake, body weight (C), systolic blood pressure (D) and Kaplan-Meier survival analysis (E) in control (Cont) and CM-treated (CM) Dahl rats. (A-D) Rats analyzed numbered 19 for both groups. Data are shown as the mean ± SEM. *P*-values indicate comparisons with control rats calculated using Bonferroni’s post hoc test in one-way ANOVA (* *P*<0.05, ** *P* <0.01). (E) Rats analyzed numbered 26 and 25 for control and CM-treated, respectively. *P*-value indicates comparison with control rats assessed using a log-rank test.

Average body weight was either clearly or gradually reduced in the control or CM-treated rats, respectively, at 11 ∼ 12 weeks of age, and thereafter was significantly reduced in the control rats compared with their CM-treated counterparts (Fig. 1). The daily food intake of both rats was drastically increased until 7 weeks of age, and remained unchanged for the subsequent 2 weeks. After a drastic reduction in food intake from 9 to 10 weeks of age, it was gradually and constantly reduced until death, although CM-treated rats were fed slightly more (Fig. 1). The daily intake was ∼25 g from 7 to 9 weeks of age, and was reduced to less than 20 g after 10 weeks of age, then lower yet to a point that was less than half of the maximum amount over time. However, the body weight increased until 11 or 12 weeks of age in the control or CM-treated rats, respectively, thereafter, and was not so drastically reduced. During this time, the body weight was maintained at ∼250 g, or more, despite being significantly less in the control rats. The daily water intake was also increased drastically until 8 weeks of age in both groups of rats, and, different from the food intake, remained almost constant until ∼14 weeks of age in the control rats and ∼18 weeks of age in the CM-treated rats. It was then drastically reduced in both groups until death (Fig. 1).

Systolic blood pressure increased gradually in both groups of rats until 11–12 weeks of age, and was slightly, albeit not significantly, higher in the control rats (Fig. 1). Systolic pressure was then reduced somewhat to around 200 mmHg or less, until death. Around 10–12 weeks of age, when blood pressure was at its maximum, both groups suffered strokes that were clearly recognized by nasal hemorrhaging with convulsions. After the brain hemorrhage, the blood pressure was not increased and tended to be unstable.

When compared with the control group, the survival of the CM-treated rats was significantly extended by ∼25 days (average survival, 86 days (∼12 weeks for control vs. 111 days (∼16 weeks) for CM) (Fig. 1). Control and CM-treated rats began to die at around 70 and 80 days of age, respectively. After ∼12 weeks of age, both groups tended to be unchanged or to decrease in weight from the reduced food intake and the unstable blood pressure, possibly due to the stroke-induced brain dysfunction or deteriorated heart failure. This period, around 12 weeks of age, was the average survival time for the control rats, but for the CM-treated rats the average survival time was approximately one month longer.

### 3.2 Light and electron microscopic analysis

#### 3.2.1 Neurons in the CNS

**Fig. 2:**
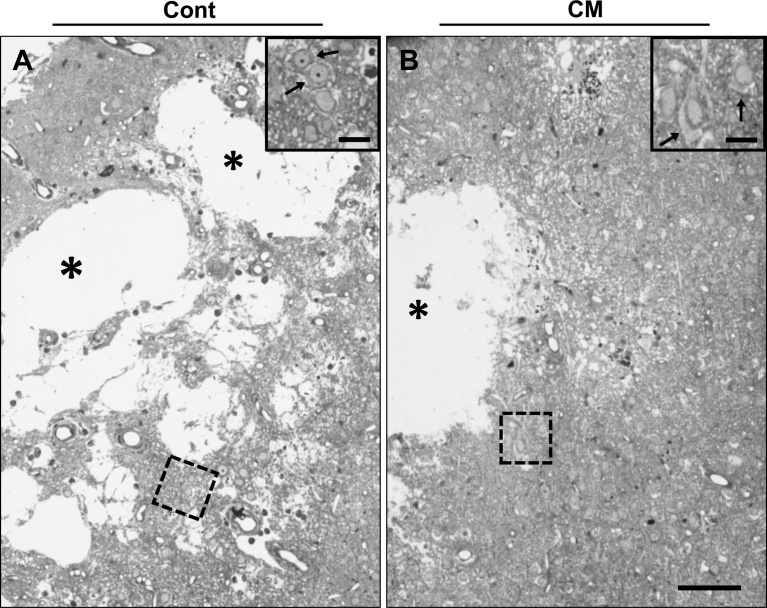
Light microscopic images of cerebral cortex in control and CM-treated rats. Lacunar-like cavities (asterisks) were visible in both control (A) and CM-treated (B) rats. Dotted squares are magnified in the insets, in which perikaryal neuronal cytoplasm (arrows) is darker and lighter in control and CM-treated rats, respectively. Scales, 100 μm and 25 μm in insets.

**Fig. 3:**
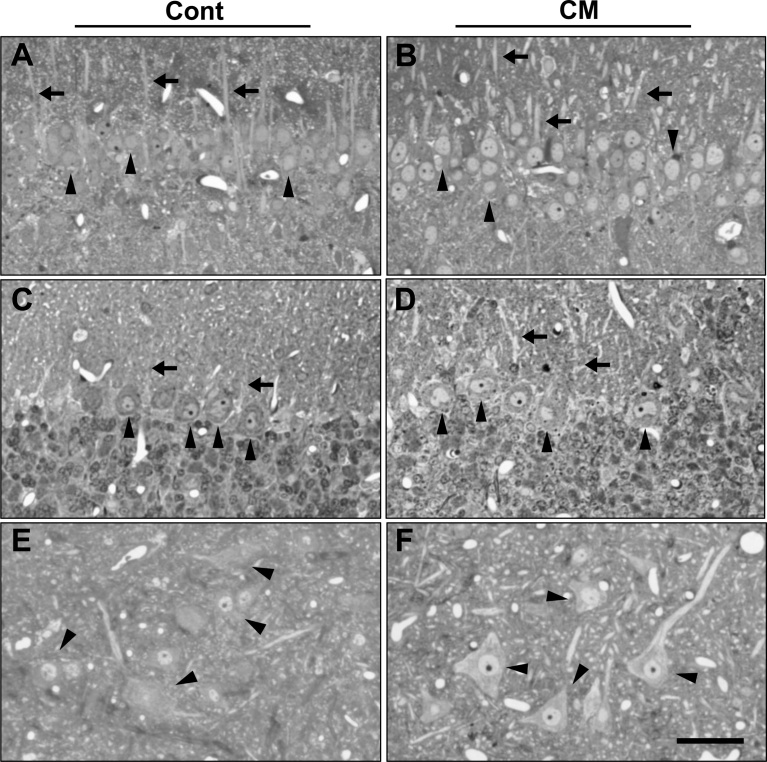
Light microscopic images of hippocampus CA1 pyramidal cells (A, B), cerebellar Purkinje cells (C, D), and spinal cord anterior horn cells (E, F) in control (A, C, E) and CM-treated (B, D, F) rats. Dendrites and cell bodies are indicated by arrows and arrowheads, respectively, in all neurons. Scale, 50 μm.

Since cerebral hemorrhage appeared mostly at around 10–12 weeks of age in both groups of Dahl rats, the CNS tissue from the rats at 10–12 weeks of age was analyzed by light microscope to determine how the neurons were influenced. As mentioned in the Animal section of Materials and methods, all data shown here and hereafter are the results of the rats at 11 weeks of age. In the control and CM-treated rat brains, lacunar-like cavities were visible in the cerebral cortex. Such cavities were the result of cerebral hemorrhage, and erythrocytes could still be seen among the tissues (Fig. 2). Neuronal cells near the cavities tended to show necrotic or apoptotic profiles and a denser cytoplasm, that is a sign of neurodegeneration (Shiozaki et al., 2008), in both groups. However, some neurons seemed somewhat normal with a lighter cytoplasm in the CM-treated rats (Fig. 2). In the hippocampal CA1, most pyramidal cells appeared either denser or lighter in the nucleoplasm and cytoplasm of either the control or CM-treated rats, respectively, and the Purkinje cells in the cerebellum showed similar differences in staining. Anterior horn cells in the spinal cord also showed similar differences between the control and CM-treated groups, but these were not as clear as those for either the hippocampal or Purkinje cells (Fig. 3).

**Fig. 4:**
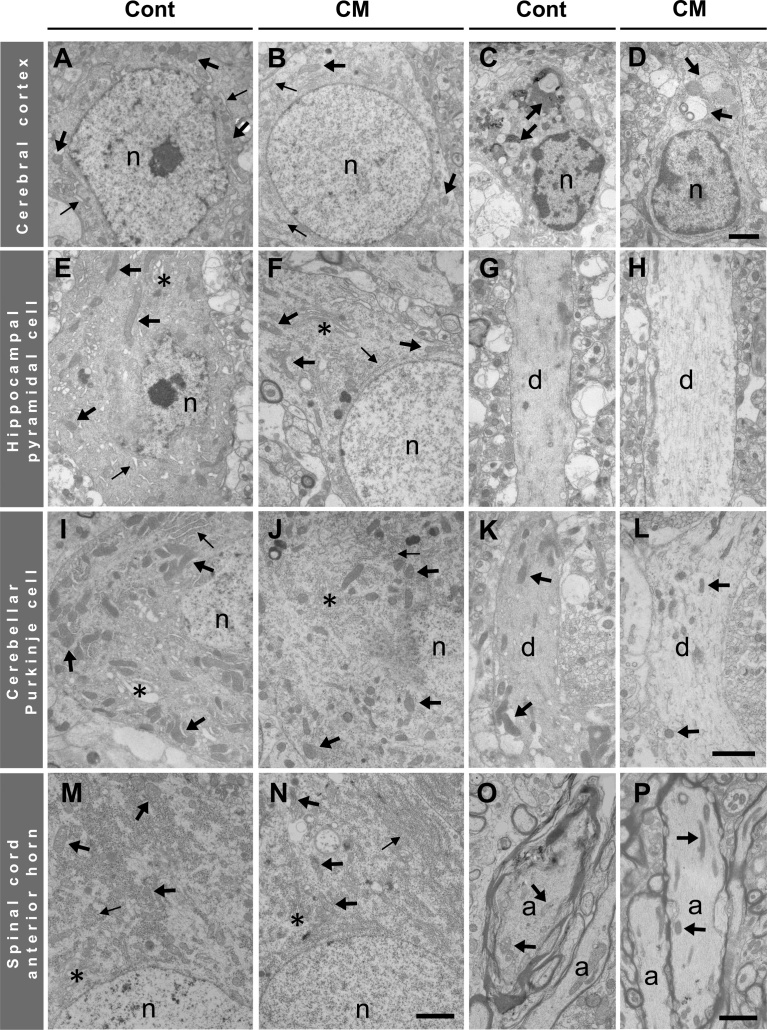
Electron micrographs of cerebral cortical neurons (A, B) and microglial cells (C, D), hipocampal pyramidal cells (E-H), cerebellar Purkinje cells (I-L), spinal cord anterior horn cells (M, N) and myelinated axons (O, P) in control (Cont) (A, C, E, G, I, K, M, O) and CM-treated rats (CM) (B, D, F, H, J, L, N, P). n, nucleus; thick arrows in A-P (except in C and E, phagosomes), mitochondria; thin arrows in A, B, E, F, I, J, M and N, rER; asterisks, Golgi apparatus; d, dendrites of pyramidal cells (G, H) and Purkinje cells (K, L); a, myelinated axons. Scales, 2 μm for A-D, and for E, F, I, J, M, N, and for G, H, K, L, and for O, P, respectively.

**Fig. 5:**
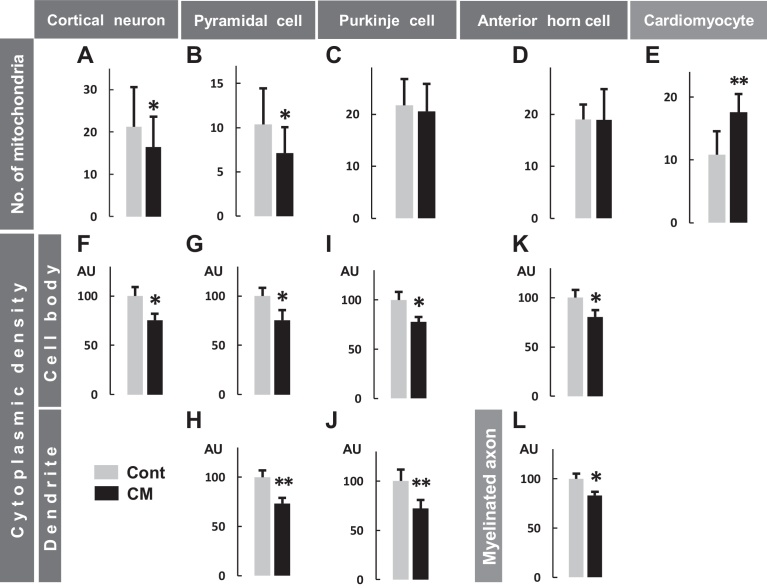
Quantification of mitochondrial number and neuronal cytoplasmic density in control (Cont) and CM-treated (CM) rats. Number of mitochondria in cortical neuronal (A), pyramidal (B), Purkinje (C) and anterior horn (D) cell bodies per 50 μm^2^ and in cardiomyocytes (E) per 20 μm in length between myofibrils. Cytoplasmic density, shown by electron micrographs, in cortical neuronal cell bodies (F), pyramidal cell bodies (G) and dendrites (H), Purkinje cell bodies (I) and dendrites (J), anterior horn cell bodies (K) and myelinated axons (L). The number of mitochondria was significantly (* *P* < 0.05) less in cortical neurons and pyramidal cells but greater (** *P* < 0.01) in cardiomyocytes in CM-treated rats. The cytoplasmic density values from the control rats were normalized using 100 as a mean value for arbitrary units (AU), and all images showed significantly (* *P* < 0.05, ** *P* <0.01) lower levels in CM-treated rats.

The above-mentioned light microscopic images were confirmed with electron microscopy. In the cerebral cortex, neurons showed apoptotic profiles with a denser cytoplasm (Figs. 4, 5), an expanded rough endoplasmic reticulum (rER) and Golgi apparatus, and chromatin condensation in control rats by comparison with those in CM-treated rats (Fig. 4). Curiously, mitochondria in cortical neurons were significantly reduced in distribution density or in number in CM-treated rats (Figs. 4, 5). Active microglial cells phagocytosing debris were frequently visible (Fig. 4) accompanied by many erythrocytes in the tissue space outside blood capillaries in both rats.

In hippocampal CA1 pyramidal and cerebellar Purkinje cells, the differences in neuronal profiles were easily compared between both groups of rats due to the simply organized configuration of these neurons, and it was clear that the dendritic and perikaryal cytoplasm was darker (Figs. 4, 5) with an expanded rER and Golgi apparatus, and the nucleus was also darker with condensed chromatin and a developed nucleolus in the control rats (Fig. 4). Mitochondria appeared to have a similar profile in both groups of rats. By comparison with the control rats, the distribution density of mitochondria was significantly decreased in the hippocampal pyramidal cells, as in cerebral cortical neurons, but not in the Purkinje cells in the CM-treated rats (Figs. 4, 5).

In the spinal cord anterior horn, where motor axons from the cerebral cortex terminate to form synapses with anterior horn cells, the anterior horn cells also showed cellular changes, basically similar to those seen in the neurons of the brain. Both the rER and the Golgi apparatus were also expanded in the control cells, but as in the Purkinje cells, the mitochondria were unchanged in number for both groups (Figs. 4, 5). Myelinated axons often showed a denser axoplasm, and there was an accumulation of organelles with degenerated profiles in the control rats (Figs. 4, 5).

#### 3.2.2 Cardiovascular system: Cardiomyocytes and aorta

**Fig. 6:**
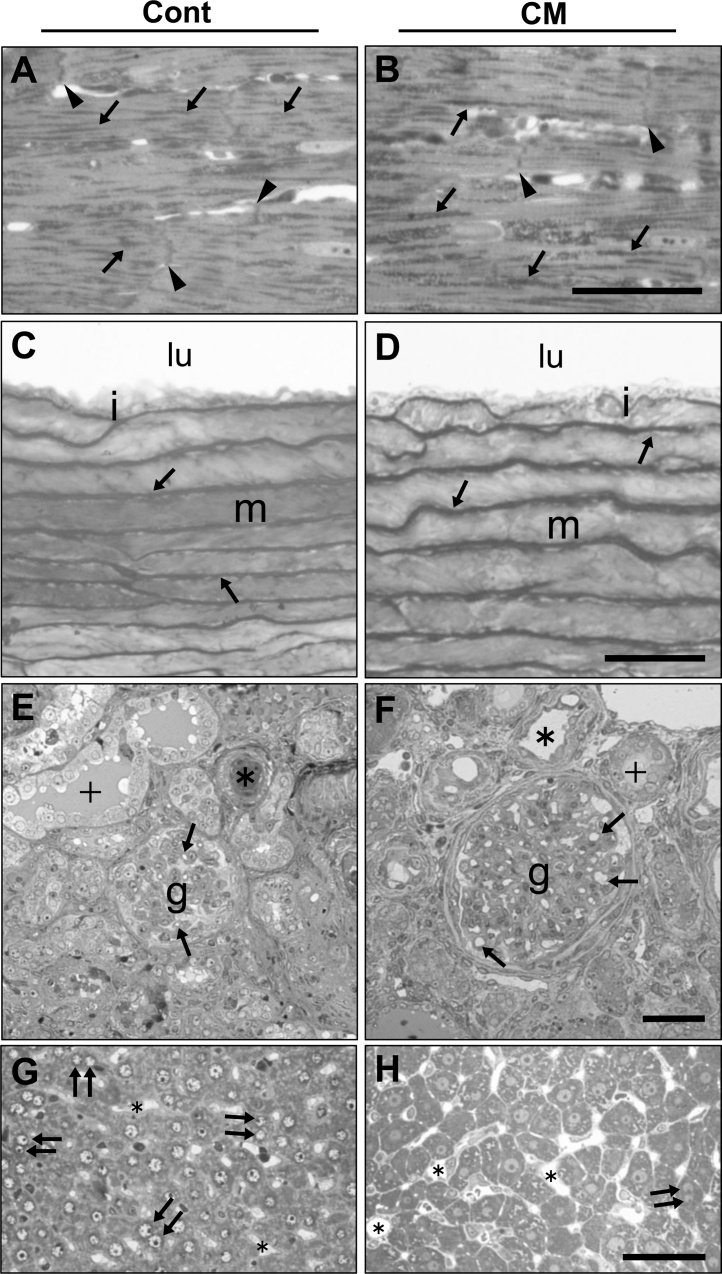
Light microscopic images of cardiomyocytes (A, B), aorta (C, D), kidney (E, F) and hepatocytes (G, H) in control (A, C, E, G) and CM-treated (B, D, F, H) rats. (A, B) arrows, densely stained linear profiles; arrowheads, intercalated disks. (C, D) lu, lumen; i, intima; m, media; arrows, elastic lamina. (E, F) arrows, glomerular (g) capillary lumens; +, renal tubules/collecting ducts; asterisks, blood vessels. (G, H) arrows, nuclei of hepatocytes more closely located; asterisks, hepatic blood capillaries. Scales, 50 μm.

**Fig. 7:**
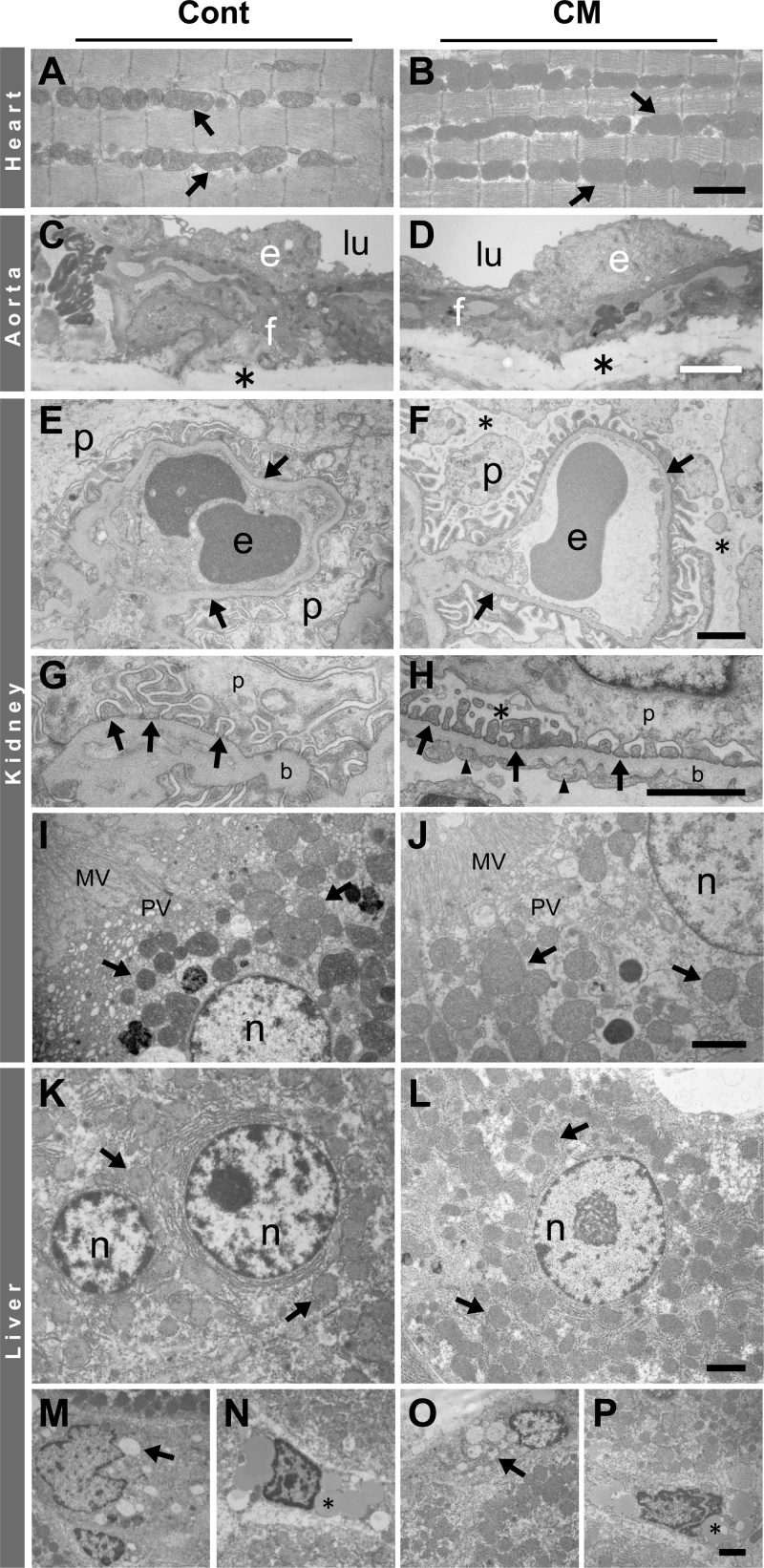
Electron micrographs of cardiomyocytes in heart (A, B), aortic tunica intima (C, D), glomeruli composed of podocytes and blood capillary (E, F, G, H) and proximal renal tubular epithelial cells (I, J) in kidney, and hepatocytes (K, L), Kupffer cells (M, O) and Ito cells (N, P) in liver. (A, B) arrows, mitochondria. (C, D) e, endothelial cells; f, fibroblasts; asterisks, elastic lamina; lu, lumen. (E, F) arrows, basement membrane; p, podocytes; e, erythrocytes in capillaries; asterisks in F, Bowman’s urinary space which is almost disappears in E. (G, H) arrows, foot processes of podocytes (p); asterisks in H, Bowman’s space which disappears in G; b, basement membrane; arrowheads in F, endothelial cells. (I, J) arrows, mitochondria; pv, pinocytotic vesicles; mv, microvilli; n, nucleus. (K, L) hepatocytes provided with two nuclei (n) in I and one nucleus (n) in J. arrows, mitochondria. (M, O) Kupffer cells with many phagosomes (arrows). (N, P) Ito cells occupied with lipid droplets (asterisks). Scales, 2 μm.

Since both groups of rats showed high blood pressure when fed a high-salt diet, we analyzed cardiomyocytes from the left ventricle in the heart and the portion of the ascending aorta. When cardiomyocytes were analyzed by light microscopy, strand-like stained profiles were detectable. These profiles, possibly corresponding to mitochondria, were more obvious in the CM-treated rats (Fig. 6). In addition to myofibrils composed of actin and myosin filaments, electron microscopy showed mitochondria as the conspicuous organelle aligned linearly between myofibrils. The mitochondrial matrix was lighter in the control group, but denser in their CM-treated counterparts (Fig. 7), which suggested that cell death-related proteins, such as cytochrome C, were released from the mitochondria to the cytoplasm and induced apoptosis in the former group (Chandel, 2014). The mitochondria appeared more regularly aligned and significantly increased in number in CM-treated rats (Figs. 5, 7).

In the ascending aorta, there was no significant difference in the vascular walls, the intima, media and adventitia, between the control and the CM-treated rats. Endothelial cells, smooth muscle cells, and the connective tissue between them also had similar profiles in both groups (Figs. 6, 7). In addition, there was no marked difference in other portions of aorta at macroscopical level (data not shown).

#### 3.2.3 Glomeruli and renal tubules in the kidney

The kidney is susceptible to hypertension and injury in Dahl rats (Khan et al., 2013). Light microscopy showed the glomerular blood capillaries had collapsed with lumens that had almost disappeared and Bowman’s urinary spaces that were cramped in the control rats, but the capillary lumens and urinary spaces were clearly detectable in the CM-treated rats (Fig. 6). The renal and collecting tubules had similar profiles in both groups, but the lumens tended to contain more amorphous substance in the control rats (Fig. 6).

Electron microscopy showed podocytes with foot processes that were irregular in width and sometimes effaced in the control rats (Fig. 7). Mitochondria in the podocytes were scanty in both rats, and frequently swollen in the control rats. In the control rats, the glomerular blood capillaries were frequently congested with erythrocytes and leukocytes (Fig. 7), as shown by the collapsed capillary images shown in light microscopy. The glomerular structures looked normal in the CM-treated rats. In the renal tubules, mitochondria were more abundant in the epithelial cells than in the podocytes, and they showed a slightly denser cytoplasmic matrix with enhanced pinocytosis and irregularly aligned microvilli in the control rats. These profiles were almost normal in the CM-treated rats (Fig. 7). The lumens of the renal tubules in the control rats were occupied with proteinaceous materials more frequently compared with the CM-treated rats (Fig. 7). It was clear that CM improved the hypertension-induced podocyte injury that causes proteinuria and results in a possible dysfunction of the renal tubules.

#### 3.2.4 Liver tissue and hepatocytes

Light microscopy showed the nuclei of hepatocytes to be located closer to each other, with conspicuous nucleoli in the control rats, which suggested that the cell division of hepatocytes was more accelerated in the control rats compared with that in the CM-treated rats. No other significant differences were seen in the hepatocytes between the two groups (Fig. 6). With electron microscopy, hepatocytes with two nuclei were detectable in both groups, but appeared to be more frequent in the control rats (Fig. 7). Further, the hepatocytic nuclei in the control group had denser chromatins and nucleoli than that found in the CM-treated rats. Other cell organelles including mitochondria had similar profiles in both groups. Kupffer cells appeared to be more active in the control rats, due to the presence of a greater number of lysosomal-associated profiles or phagosomes. Ito cells contained many more lipid droplets in the control rats (Fig. 7).

### 3.3 Immunoblot analysis

#### 3.3.1 CNS tissues: brain and spinal cord

**Fig. 8:**
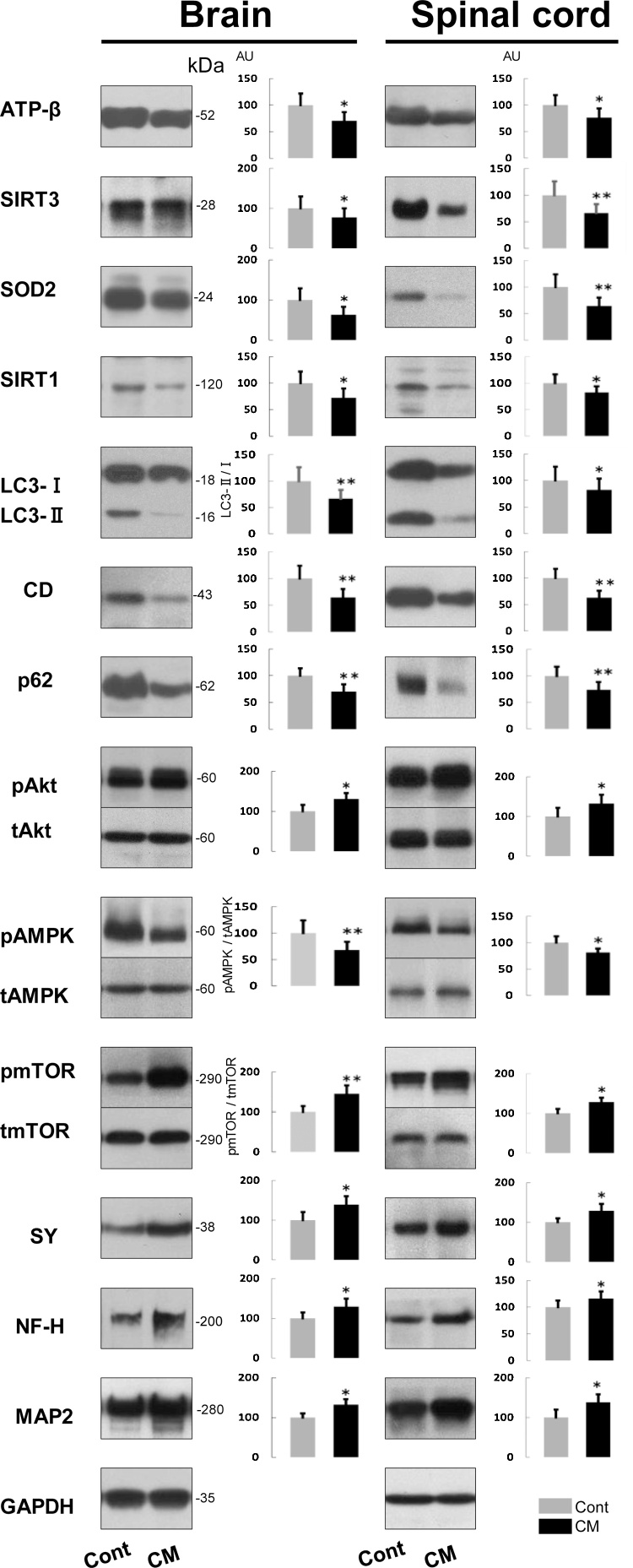
Immunoblots and their quantification of mitochondria-, autophagy- and cell-survival-related and neuron-specific proteins expressed in the CNS (brain and spinal cord) of control (Cont) and CM-treated (CM) rats. CD, cathepsin D; SY, synaptophysin. Immunoblot signal intensity was normalized to GAPDH unless otherwise mentioned, in which LC3-II/LC3-I, p (phosphorylated) Akt/t (total) Akt, pAMPK/tAMPK, pmTOR/tmTOR ratios are shown. Values from control rats are expressed as a mean value of 100 in arbitrary units (AU). Significant differences in expression or ratio in CM-treated rats compared with that in control rats are shown as * P< 0.05, ** P< 0.01. Full, uncropped images of blots are available as Supplementary Material (Figs. S8.1,2).

Neuron-specific and cell survival-related proteins including those related to mitochondria and autophagy showed basically the same expression in the brain and spinal cord, and, therefore, we combined the results from two CNS portions here. Neuron-specific proteins, microtubule-associated protein 2 (MAP2), neurofilament H protein (NF-H, nonphosphorylated form), and synaptophysin were significantly increased in expression in CM-treated rats by comparison with control rats (Fig. 8), which indicated that the neurons were either more active or stabilized as a result of the CM treatment.

The expressions of proteins, including mitochondria-, autophagy- and cell survival-related proteins, were analyzed to discern how they might have been altered by CM supplementation. The following results are from the CM-treated rats in comparison with the control rats. Mitochondria-associated proteins, the mitochondrial ATP synthase β subunit (ATP-β), SIRT3, and SOD2 were all significantly reduced in expression (Fig. 8), which may correspond to reduced number of mitochondria shown in cerebral neurons including hippocampal ones, despite no change in their number in Purkinje and anterior horn cells. SIRT1 is another sirtuin family protein that is located in both the nucleus and the cytoplasm, and it also was significantly reduced in expression. These results suggested that the mitochondrial activity had declined with CM treatment. Concerning autophagy-related proteins, the LC3-II/LC3-I ratio and cathepsin D expression both were significantly reduced (Fig. 8). However, it is curious that p62, an autophagy substrate, was significantly reduced in expression (Fig. 8). Although the reduced expression of p62 with a simultaneous reduction of the LC3-II/LC3-I ratio could be associated with increased autophagy (Klionsky et al., 2016), it would not be possible to unequiviocally state here that autophagic functioning was upregulated by CM.

Due to the possible inactivation of mitochondria with an ambiguous autophagic function, we next analyzed cell survival- or death-related proteins that may regulate mitochondria and/or autophagy functions (Fig. 8). Akt activity was assessed according to its phosphorylation level, because it is assumed to be related to cell survival via the regulation of autophagy (Xu et al., 2014). The ratio of pAkt/Akt was significantly increased, which indicated that AKt was activated. The activity of AMPK, regulating cellular energy metabolism and promoting mitochondrial biogenesis (Dasgupta and Milbrandt, 2007), was also assessed by its degree of phosphorylation, which is also related to Akt (Xu et al., 2014), but possibly functions differently in autophagy (Hardie, 2011; Kim et al., 2011). The pAMPK/AMPK ratio was significantly reduced, indicating that AMPK activity was suppressed. The activity of mTOR, an essential negative regulator of autophagy along with Akt (Ejaz et al., 2016; Wang et al., 2017) and also related to AMPK on direct modulation of Ulk1, the autophagy initiating kinase (Kim et al., 2011), was analyzed by changes in the ratio of pmTOR/mTOR. The pmTOR/mTOR ratio was significantly enhanced, showing that mTOR was activated. Although possible activations of mTOR together with Akt might lead to the suppression of autophagy in the CNS, the reduced expression of p62 seems to counter this idea.

#### 3.3.2 Heart, kidney and liver

**Fig. 9:**
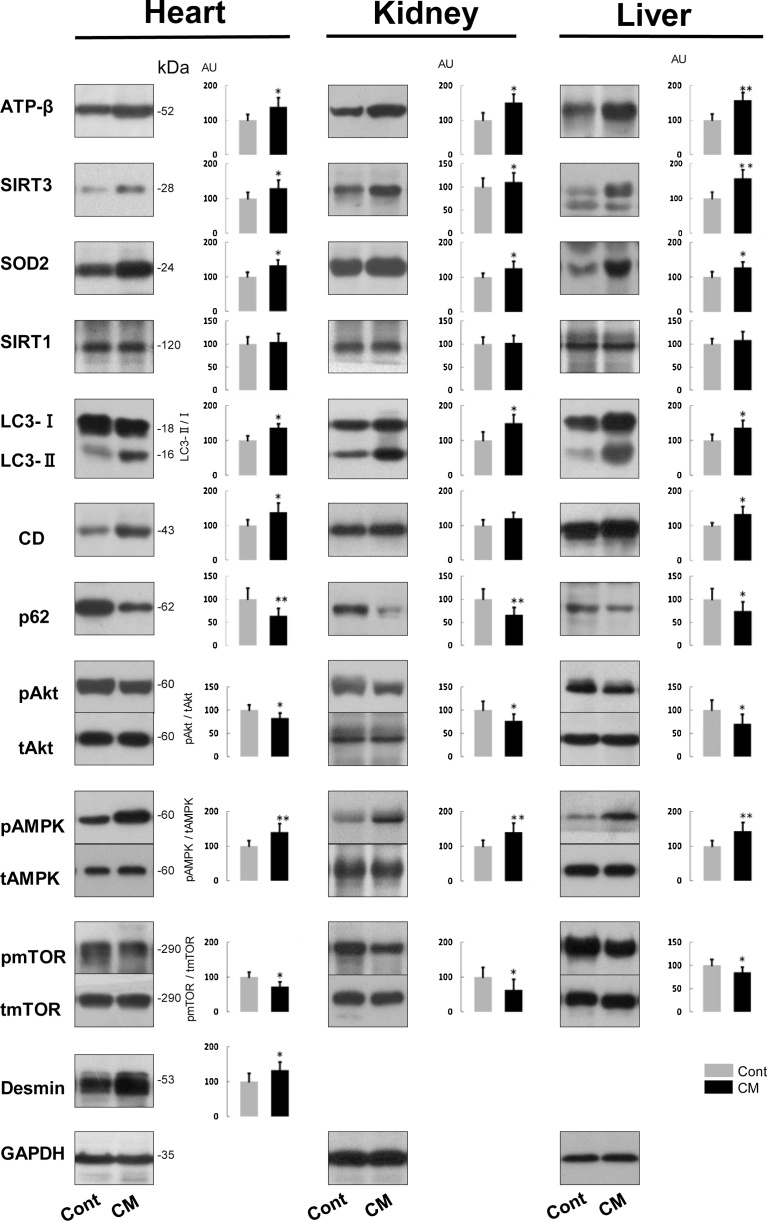
Immunoblots and their quantification of mitochondria-, autophagy- and cell-survival-related and muscle-specific (desmin) proteins expressed in heart, kidney and liver of control (Cont) and CM-treated (CM) rats. CD, cathepsin D. Blot signal intensity was normalized to GAPDH unless otherwise mentioned, in which LC3-II/LC3-I, pAkt/tAkt, pAMPK/tAMPK, and pmTOR/tmTOR ratios are shown. Values from control rats are expressed as a mean value of 100 in arbitrary units (AU). Significant differences in expression or ratio in CM-treated rats compared with that in control rats are shown as * P< 0.05, ** P< 0.01. Full, uncropped images of blots are available as Supplementary Material (Figs.S9.1–3).

These three organs showed similar results in their expressions of mitochondria-, autophagy- and cell survival-related proteins, and, thus, their results are described together. Compared with the control rats, the results from CM-treated rats were as follows. ATP-β, SIRT3 and SOD2 were significantly increased in expression (Fig. 9). These results supported the concept whereby mitochondria are enhanced in function in all three organs, despite the lack of significant change in the expression of SIRT1 (Fig. 9). The LC3-II/LC3-I ratio and cathepsin D expression were increased significantly, except kidney’s cathepsin D that was just slightly increased (Fig. 9). Corresponding to these changes, p62 was significantly reduced in expression (Fig. 9), suggesting that autophagic function was accelerated. The ratio of pAkt/Akt was significantly decreased (Fig. 9), which indicated that Akt activity had been suppressed. The pAMPK/AMPK ratio was significantly increased (Fig. 9), indicating that AMPK activity had been enhanced. The pmTOR/mTOR ratio was significantly reduced in all organs (Fig. 9), meaning mTOR activity was repressed. These biochemical results suggested that autophagy had been activated in these extra-CNS organs because of the treatment with CM.

### 3.4 Immunohistochemical analysis

#### 3.4.1 CNS (cerebellar Purkinje cell and spinal cord anterior horn cell)

**Fig. 10:**
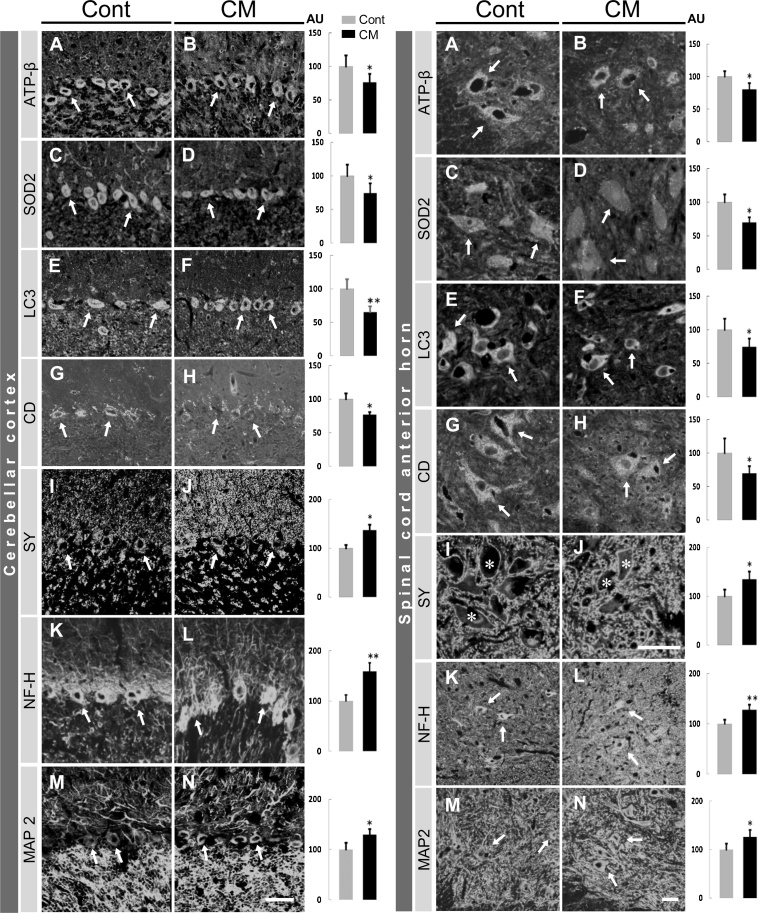
Immunohistochemically labeled images in cerebellar cortex (left) and spinal cord anterior horn (right) with quantification in control (Cont) and CM-treated (CM) rats. The value for fluorescent intensity of the control is expressed as a mean of 100 in arbitrary units (AU). CD, cathepsin D; SY, synaptophysin. In the cerebellar cortex, arrows in A-N indicate Purkinje cells. In the spinal cord anterior horn, arrows in A-N, except I and J, indicate anterior horn cells which are marked by asterisks. Significant differences in expression in CM-treated rats compared with that in control rats are shown as * *P* <0.05, ** *P* <0.01. Scales, 50 μm in A-N in the cerebellar cortex, in A-J and K-N in the spinal cord anterior horn.

In the CNS, we selected Purkinje cells in the cerebellum and anterior horn cells in the spinal cord, which facilitated comparison of the same kinds of neurons between control and CM-treated rats via confocal microscope. By comparison with control rats, the changes in immunolabeled intensity among the CM-treated rats were as follows. The mitochondria-related proteins ATP-β and SOD2 were significantly reduced in labeling intensity in Purkinje cells and in anterior horn cells (Fig. 10), despite no significant differences in the mitochondrial number of these cells when analyzed by electron microscopy. Such differences could amount to differences in the size of the mitochondria, which appeared to be smaller in CM-treated rats (Fig. 4). In addition, immunoreactivity was predominantly restricted in the neurons but not in glial cells. The difference in immunolabeling intensity corresponded well with the results obtained from immunoblotting for the brain and spinal cord tissues. The labeling of autophagy-related proteins LC3 and cathepsin D were also significantly reduced in these neurons. Although these results suggested that the mitochondrial and autophagic functions of the CNS neurons had declined, we could not preclude the counter possibility that autophagy had been enhanced, due to the lack of specific immunostaining for p62.

The labeling intensity for neuron-specific proteins synaptophysin, MAP2 and NF-H, as shown by the immunoblotting, was increased significantly in the cerebellar cortex and in the spinal cord anterior horn. Synaptophysin immunolabeling, localized in the axon terminals, was enhanced particularly around the anterior horn cells (Fig. 10). The labeling of MAP2, localized in the neuronal cell bodies and dendrites, was enhanced significantly in the Purkinje cells and in the anterior horn cells (Fig. 10). NF-H labeling was also enhanced significantly in and around Purkinje cells and in the anterior horn cells (Fig. 10). The elevated expressions of these neuron-specific proteins indicated that the CNS neurons were vigorous but stable in nature following CM treatment (Shiozaki et al., 2008).

#### 3.4.2 Heart, kidney and liver

**Fig. 11:**
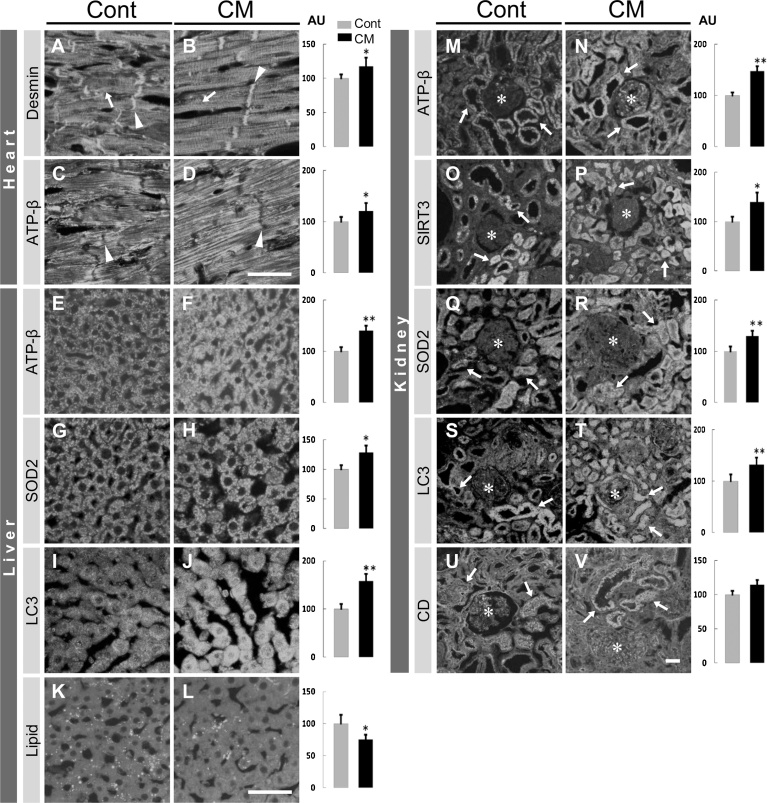
Immunolabeled images in heart, liver (left) and kidney (right) and lipid staining in liver (left) with quantitative analysis for their labeling intensity in control (Cont) and CM-treated (CM) rats. (A-D) Arrowheads and arrows indicate intercalated disks and Z bands, respectively, in cardiomyocytes. In the liver (E-L), immunostaining with the respective antibodies ((E-J) and lipid staining (K, L). In the kidney (M-V), epithelial cells of renal tubules (arrows) are labeled, but glomerular structures (asterisks) are not significantly labeled. CD, cathepsin D. Values from control rats are expressed as a mean value of 100 in arbitrary units (AU). Significant differences in expression in CM-treated rats compared with that in control rats are shown as * *P* < 0.05, ** *P* <0.01. Scales, 50 μm in A-D for the heart, in E-L for the liver, in M-V for the kidney.

The following immunohistochemical results are from the CM-treated rats in comparison with the control rats. In the heart, the labeling for desmin, a muscle-specific intermediate filament protein, was restricted to intercalated disks and Z lines in cardiomyocytes, and was significantly increased in intensity (Fig. 11). We could not specifically label the mitochondria- and autophagy-related proteins, except ATP-β that was enhanced significantly in reactivity (Fig. 11), in the cardiomyocytes.

In the kidney, immunoreactivity for mitochondria-related proteins ATP-β, SIRT3 and SOD2 was predominantly restricted to the epithelial cells of the renal tubules and scarcely in the glomeruli (Fig. 11), which may have corresponded to abundant and scant mitochondria in the renal tubular epithelial cells and podocytes, respectively, in both groups. All three mitochondrial-related proteins were significantly increased in labeling intensity in the renal tubules (Fig. 11). Autophagy-related proteins LC3 and cathepsin D were also enhanced, but not significant for cathepsin D, in labeling intensity in the renal tubules (Fig. 11). These proteins were almost undetectable in the glomeruli. Although the mitochondria and autophagy functions in the epithelial cells of renal tubules appeared to be activated by CM, these functions were not likely to be related to the filtration function of podocytes.

In the liver, the labeling of ATP-β and SOD2 was increased significantly in hepatocytes. These immuno-positive dots appeared to correspond in size and distribution in the mitochondria (Fig. 11), which suggested that the mitochondria were enhanced in number and also in activity. An autophagy-related protein, LC3, was also significantly increased in intensity, suggesting that, although cathepsin D was not specifically labeled, autophagic function was enhanced. In addition, lipids in the liver were stained, and appeared to be predominantly localized in the Ito cells, and were significantly less prominent following treatment with CM (Fig. 11).

## 4 Discussion

In the present study, we have shown that treatment with CM significantly extended the survival of Dahl salt-sensitive rats, whose lifespan was shortened by a high-salt diet. The high-salt-induced short lifespans of the Dahl rats could have been caused by either hypertension-induced heart failure or stroke, and an extension of their lifespan was suggested by the delay in diastolic heart failure following treatment with another dietary compound, spermidine (Eisenberg et al., 2016). However, in our study CM supplementation did not significantly reduce the blood pressure of the Dahl rats, and stroke occurred in both control and CM-treated groups. Kidney protection of these hypertensive Dahl rats by epoxyecosatrienoic acid or natural tetrapeptide, which included anti-inflammatory or −oxidative activity, was also reported without a reduction in blood pressure (Khan et al., 2013; Worou et al., 2015). After the occurrence of a stroke, however, the behavior of the CM-treated rats tended to be unambiguously ameliorated, while control rats appeared to continue suffering from sequela of the stroke. CM is expected to improve the functions of the CNS neurons and also those of the cells of the other organs such as heart, kidney and liver, and resulting in improved health possibly via the maintenance of whole-body homeostasis. The Dahl rats reported by Eisenberg et al. (2016) were taken off a high-salt (8% NaCl) diet at 7 weeks of age, but our Dahl rats had begun feeding 2 weeks earlier and died much earlier than their rats, although the change in lifespan was not mentioned in their study. Although the possibility of heart failure could not be excluded as a cause of death, we concluded that our Dhal rats died from the complications of stroke.

Morphological analysis showed that with CM treatment all organs we analyzed were substantially ameliorated at the tissue and cellular levels, particularly in heart and kidney. However, surprisingly, biochemical analysis showed that in the CNS tissue mitochondria- and autophagy-related proteins were reduced in expression following treatment with CM. At this point, the AMPK activity was also reduced, together with reduced SIRT1 expression, while Akt and mTOR activity was enhanced, suggesting that mitochondrial and autophagic functions had been repressed in the CNS (Shiozaki et al., 2011; Price et al., 2012; Hayakawa et al., 2013; Xu et al., 2014). This may be related to the neurodegeneration that could result in either cell death or apoptosis (Maiese, 2016). As for autophagy, our data show possible activations of mTOR and Akt along with inactivation of AMPK, which is widely believed to suppress autophagy (Hardie, 2011; Roach, 2011; Kim et al., 2011 Ejaz et al., 2016; Wang et al., 2017). In addition, the inhibition of AMPK activity was thought to suppress mitophagy via preventing the translocation of Ulk1 to mitochondria (Tian et al., 2015).

However, it is an important aspect that p62 was reduced significantly in expression following treatment with CM. The downregulation of p62 along with fewer expressions of LC3-II, an active form of LC3, and lysosomal enzymes, such as cathepsin D, suggests that autophagy has been activated in CM-treated CNS following stroke due to the elimination of autophagosomes by lysosomes (Klionsky et al., 2016). P62 is a key regulator of the mTORC1 pathway and its reduction might be related to the activation of autophagy (Duran et al., 2011; Manley et al., 2013; Pun and Park, 2017). Further, p62 is considered a marker of autophagy flux (Pun and Park, 2017) that may be accelerated with reduced expressions of autophagy-related proteins including LC3-II together with elevated autophagic clearance of ubiquitinated protein accumulation (Wang et al., 2017). Therefore, it will be necessary to analyze in vivo autophagic flux using autophagy or lysosome inhibitors before deciding if autophagy is upregulated in the CM-treated CNS. Although mitophagy was not analyzed here, there is a possibility that depolarized mitochondria, marked by a PINK1-Parkin-Mfn2-dependent mechanism, might be degraded by mitophagy (Chen and Dorn, 2013). The increased mitophagy may correspond to fewer expressions of mitochondria-related proteins and a lessening of the mitochondrial number. It is thus possible that autophagy including mitophagy must be accelerated in the CM-treated CNS in a mechanism that differs from other organs.

The accumulation of metabolically harmful substances tagged with p62 in neurons may be eliminated by a unique form of CM-induced autophagy in the CNS. This may correspond to the suppression of cellular damage that induces apoptosis in the nervous system (Chong et al., 2012) and be involved in protecting neurons against either cerebral hemorrhage or ischemia (Gubern et al., 2014). With CM treatment, the CNS neurons thus appeared to be stabilized or ameliorated in function, with possible reduced mitochondria but activated autophagy (Zhao et al., 2016), as demonstrated by repressed AMPK activity caused by a decline in cellular energy metabolism. Such reduced energy expenditure in neurons appeared to be caused either by caloric restriction (Schafer et al., 2015) or by nutrient deprivation (Yang et al., 2013), which is critical for neuronal survival and/or prevention against stroke-induced deterioration of the CNS milieu (Yang et al., 2013).

In the heart, kidney and liver, which are essential for maintaining metabolic homeostasis, mitochondrial and autophagic functions must be activated due to the increased expressions of mitochondrial proteins (ATP-β, SIRT3 and SOD2) and enhanced AMPK activity but reduced Akt and mTOR activities (Tian et al., 2015; Zhang et al., 2017). Since autophagy is enhanced in these organs in a well-accepted manner and different from that proposed in the CNS, the mitophagic machinery may also be activated (Egan et al., 2011), but mitochondria appear to increase in number and activity. This is because dietary antiaging phytochemicals, such as CM, are suggested to promote health and extend lifespan in various animal models via multiple mechanisms including the induction of autophagy as well as promoting mitochondrial function. Interestingly, such proposed antiaging effects of phytochemicals are suggested to be shared by caloric restriction (Hayakawa et al., 2013; Si and Liu, 2014). We thus speculate that activations of autophagy and mitochondria are independent of each other, or that degradation of dysfunctional mitochondria by mitophagy is accompanied by the simultaneous elevation of CM-mediated mitochondrial biogenesis, possibly resulting in increases in mitochondria (Dasgupta and Milbrandt, 2007; Hayakawa et al., 2013; Andres et al., 2017).

Diastolic heart failure is thought to be the cause of death in Dahl rats (Eisenberg et al., 2016), and our microscopic analysis also suggested that the cardiomyocytes in these control rats were dysfunctional due to reduced mitochondria and autophagy functions. However, such hypertension-induced heart failure was improved with CM treatment, albeit curiously without a significant reduction in blood pressure. In such CM-treated Dahl rats, mitochondria and autophagy must be activated, which results in protective measures for the circulatory system in order to maintain homeostasis in vivo by providing oxygen and other nutrients throughout the body. In the kidney, according to morphological analysis, glomerular function must be significantly ameliorated by treatment with CM (Khan et al., 2013; Yu et al., 2016), although such improvement might be associated with the cascade that seems to be activated by CM without going through the function of either mitochondria or autophagy. Immunohistochemical analysis suggests that the mitochondria and autophagy functions are enhanced in renal tubular epithelial cells, instead of in glomerular podocytes, and CM may ameliorate the function of renal tubules via the activation of mitochondria and autophagy. The improvement in renal functions is important for eliminating waste/toxic metabolites from the blood in order to maintain in whole-body homeostasis. Concerning the function of the liver, which is central to metabolic regulation (Shiozaki et al., 2011), although a hypertension-induced morphological change was not detectable in hepatocytes, despite a slight activation of Kupffer and Ito cells in the control rats, biochemical and immunohistochemical analysis strongly suggests that mitochondrial and autophagic functions are improved by treatment with CM.

## 5 Conclusion

**Fig. 12:**
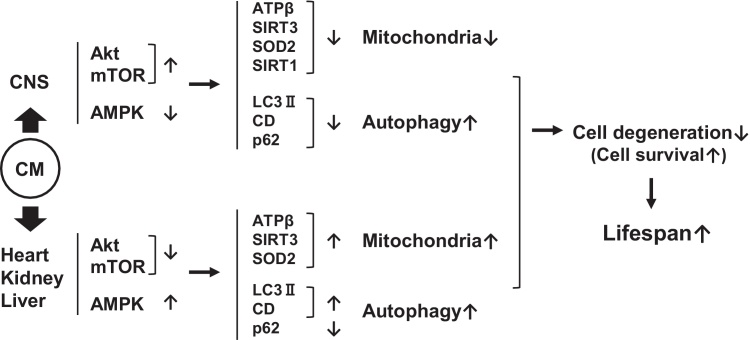
Possible pathways for the improvement of survival by *Cordyceps militaris* (CM) in stroke-caused Dahl salt-sensitive hypertensive rats through modulating mitochondria and autophagy functions in the CNS and the other three organs. CD, cathepsin D.

CM was found to improve whole-body health through direct influences on cellular, tissue and organ levels with alterations in mitochondria- autophagy- and cell survival- protein expressions. CM may also be associated with the protection of the CNS by the extra-CNS organs following cerebral hemorrhage via the provision of blood rich in nutrients with lower levels of waste products. The CNS neurons may require less metabolic energy to support their functions, possibly with the assistance of activated peripheral organs. Interestingly, mitochondria and autophagy functions appeared to be quite different in manner between the CNS and other peripheral organs when treated with CM. In all the peripheral organs, both mitochondria and autophagy were activated. However, in the CNS, mitochondria were less active and autophagy appeared to be activated but in a way that differed from that shown in the peripheral organs. The activation of autophagy may be necessary in the CNS for maintaining neuronal homeostasis including mitochondrial quality, although further studies are necessary to clarify whether autophagy and mitophagy are actually upregulated in this organ. Such organ-dependent cellular activation and inactivation might be related to an extension of the survival of life-shortened Dahl rats (Fig. 12). Lifespan extension effect of CM shown in these rats should be confirmed in normal mammals, providing that this mushroom is recommended for therapeutic approaches to age- or hypertension-related diseases.

## Declarations

### Author contribution statement

Kentaro Takakura, Shogo Ito, Junya Sonoda: Performed the experiments; Analyzed and interpreted the data.

Koji Tabata: Performed the experiments.

Motoko Shiozaki, Kaoru Nagai, Masahiro Shibata, Masato Koike, Yasuo Uchiyama: Contributed reagents, materials, analysis tools or data.

Takahiro Gotow: Conceived and designed the experiments; Performed the experiments; Analyzed and interpreted the data; Wrote the paper.

### Funding statement

This work was supported in part by a Grant-in-Aid for Scientific Research (C) (No. 21590225 to T.G.), Japan Society for the Promotion of Science (JSPS).

### Competing interest statement

The authors declare no conflict of interest.

### Additional information

Supplementary content related to this article has been published online at http://dx.doi.org/10.1016/j.heliyon.2017.e00462.

No additional information is available for this paper.

## Appendix A Supplementary data

Fig. S8.1 Brain. Full scans of original blots for data shown in Fig. 8. Panels corresponding to the images in the paper are indicated. CD, cathepsin D; SY, synaptophysin; C, Control rats; M, *Cordyceps militaris* (CM)-treated rats.

Fig. S8.2 Spinal cord. Full scans of original blots for data shown in Fig. 8. Panels corresponding to the images in the paper are indicated. CD, cathepsin D; SY, synaptophysin; C, Control rats; M, *Cordyceps militaris* (CM)-treated rats.

Fig. S9.1 Heart. Full scans of original blots for data shown in Fig. 9. Panels corresponding to the images in the paper are indicated. CD, cathepsin D; C, Control rats; M, *Cordyceps militaris* (CM)-treated rats.

Fig. S9.2 Kidney. Full scans of original blots for data shown in Fig. 9. Panels corresponding to the images in the paper are indicated. CD, cathepsin D; C, Control rats; M, *Cordyceps militaris* (CM)-treated rats.

Fig. S9.3 Liver. Full scans of original blots for data shown in Fig. 9. Panels corresponding to the images in the paper are indicated. CD, cathepsin D; C, Control rats; M, *Cordyceps militaris* (CM)-treated rats.

Table S1 The ARRIVE Guidelines Checklist.

## References

[bib0005] Andres A.M., Tucker K.C., Thomas A., Taylor D.J.R., Sengstock D., Salik M., Jahania S.M., Dabir R., Pourpirali S., Brown J.A. (2017). Mitophagy and mitochondrial biogenesis in atrial tissue of patients undergoing heart surgery with cardiopulmonary bypass. JCI Insight..

[bib0010] Chandel N.S. (2014). Mitochondria as signaling organelles. BMC Biol..

[bib0015] Chen Y., Dorn G.W. (2013). PINK1-phosphorylated mitofusin 2 is a Parkin receptor for culling damaged mitochondria. Science.

[bib0020] Chong Z.Z., Shang Y.C., Wang S., Maiese K. (2012). A critical kinase cascade in neurological disorder. PI 3-K. Akt, and mTOR. Future Neurol..

[bib0025] Dasgupta B., Milbrandt J. (2007). Resveratrol stimulates AMP kinase activity inneurons. Proc. Natl. Acad. Sci. USA.

[bib0030] Duran A., Amanchy R., Linares J.F., Joshi J., Abu-Baker S., Porollo A., Hansen M., Moscat J., DiazMeco M.T. (2011). p62 is a key regulator of nutrient sensing in the mTORC1 pathway. Mol. Cell.

[bib0035] Egan D.F., Shackelford D.B., Mihaylova M.M., Gelino S., Kohnz R.A., Mair W., Vasquez D.S., Joshi A., Gwinn D.M., Taylor R. (2011). Phosphorylation of ULK1 (hATG1) by AMP-activated protein kinase connects energy sensing to mitophagy. Science.

[bib0040] Eisenberg T., Knauer H., Schauer A., Büttner S., Ruckenstuhl C., Carmona-Gutierrez D., Ring J., Schroeder S., Magnes C., Antonacci L. (2009). Induction of autophagy by spermidine promotes longevity. Nat. Cell Biol..

[bib0045] Eisenberg T., Abdellatif M., Schroeder S., Primessnig U., Stekovic S., Pendl T., Harger A., Schipke J., Zimmermann A., Schmidt A. (2016). Cardioprotection and lifespan extension by the natural polyamine spermidine. Nat. Med..

[bib0050] Ejaz A., Mitterberger M.C., Lu Z., Mattesich M., Zwierzina M.E., Hörl S., Kaiser A., Viertler H.P., Rostek U., Meryk A. (2016). Weight loss upregulates the small GTPase DIRAS3 in human white adipose progenitor cells, which negatively regulates adipogenesis and activates autophagy via Akt-mTOR Inhibition. EBioMedicine.

[bib0055] Gubern C., Camós S., Hurtado O., Rodríguez R., Romera V.G., Sobrado M., Cañadas R., Moro M.A., Lizasoain I., Serena J. (2014). Characterization of Gcf2/Lrrfip1 in experimental cerebral ischemia and its role as a modulator of Akt, mTOR and β-catenin signaling pathways. Neuroscience.

[bib0060] Hardie D.G. (2011). AMPK and autophagy get connected. EMBO J..

[bib0065] Hayakawa N., Shiozaki M., Shibata M., Koike M., Uchiyama Y., Matsuura N., Gotow T. (2013). Resveratrol affects undifferentiated and differentiated PC12 cells differently: particularly with respect to possible differences in mitochondrial and autophagic functions. Eur. J. Cell Biol..

[bib0070] Hwang I.K., Lim S.S., Yoo K.Y., Lee Y.S., Kim H.G., Kang I.J., Kwon H.J., Park J., Choi S.Y., Won M.H. (2008). A phytochemically characterized extract of *Cordyceps militaris* and cordycepin protect hippocampal neurons from ischemic injury in gerbils. Planta. Med..

[bib0075] Jin M.L., Park S.Y., Kim Y.H., Oh J.I., Lee S.J., Park G. (2014). The neuroprotective effects of cordycepin inhibit glutamate-induced oxidative and ER stress-associated apoptosis in hippocampal HT22 cells. Neurotoxicol..

[bib0080] Khan A.H., Neckar J., Manthati V., Errabelli R., Pavlov T.S., Staruschenko A., Falck J.R., Imig J.D. (2013). An orally active epoxyeicosatrienoic acid analog attenuates kidney injury in hypertensive Dahl salt sensitive rat. Hypertension.

[bib0085] Kim J., Kundu M., Viollet B., Guan K.L. (2011). AMPK and mTOR regulate autophagy through direct phosphorylation of Ulk1. Nat. Cell Biol..

[bib0090] Klionsky D.J. (2016). Guidelines for the use and interpretation of assays formonitoring autophagy (3rd edition). Autophagy.

[bib0095] Li X.T., Li H.C., Li C.B., Dou D.Q., Gao M.B. (2010). Protective effects on mitochondria and anti-aging activity of polysaccharides from cultivated fruiting bodies of *Cordyceps militaris*. Am. J. Chin. Med..

[bib0100] Liu C., Song J., Teng M., Zheng X., Li X., Tian Y., Pan M., Li Y., Lee R.J., Wang D. (2016). Antidiabetic and antinephritic activities of aqueous extract of *Cordycepsmilitaris* fruit body in diet-streptozotocin-induced diabetic Sprague Dawley rats. Oxid. Med. Cell. Longev..

[bib0105] Ma L., Zhang S., Du M. (2015). Cordycepin from *Cordyceps militaris* prevents hyperglycemia in alloxan-induced diabetic mice. Nutr. Res..

[bib0110] Madeo F., Zimmermann A., Maiuri M.C., Kroemer G. (2015). Essential role forautophagy in life span extension. J. Clin. Invest..

[bib0115] Maiese K. (2016). Targeting molecules to medicine with mTOR: autophagy and neurodegenerative disorders. Br. J. Clin. Pharmacol..

[bib0120] Manley S., Williams J.A., Ding W.X. (2013). Role of p62/SQSTM1 in liver physiology and pathogenesis. Exp. Biol. Med. (Maywood).

[bib0125] Morselli E., Mariño G., Bennetzen M.V., Eisenberg T., Megalou E., Schroeder S., Cabrera S., Bénit P., Rustin P., Criollo A. (2011). Spermidine and resveratrol induce autophagy by distinct pathways converging on the acetylproteome. J. Cell Biol..

[bib0130] Park J.M., Lee J.S., Lee K.R., Ha S.J., Hong E.K. (2014). Cordyceps militaris extract protects human dermal fibroblasts against oxidative stress-induced apoptosis and premature senescence. Nutrients.

[bib0135] Price N.L., Gomes A.P., Ling A.J., Duarte F.V., Martin-Montalvo A., North B.J., Agarwal B., Ye L., Ramadori G., Teodoro J.S. (2012). SIRT1 is required for AMPK activation and the beneficial effects of resveratrol on mitochondrial function. Cell. Metab..

[bib0140] Pun N.T., Park P.H. (2017). Role of p62 in the suppression of inflammatory cytokine production by adiponectin in macrophages: Involvement of autophagy and p21/Nrf2axis. Sci. Rep..

[bib0145] Roach P.J. (2011). AMPK → ULK1 → Autophagy. Mol. Cell Biol..

[bib0150] Sanz A. (2016). Mitochondrial reactive oxygen species: Do they extend or shortenanimal lifespan?. Biochim. Biophys. Acta.

[bib0155] Schafer M.J., Dolgalev I., Alldred M.J., Heguy A., Ginsberg S.D. (2015). Calorie restriction suppresses age-dependent hippocampal transcriptional signatures. PLoS One.

[bib0160] Shiozaki M., Yoshimura K., Shibata M., Koike M., Matsuura N., Uchiyama Y., Gotow T. (2008). Morphological and biochemical signs of age-related neurodegenerative changes in klotho mutant mice. Neuroscience.

[bib0165] Shiozaki M., Hayakawa N., Shibata M., Koike M., Uchiyama Y., Gotow T. (2011). Closer association of mitochondria with lipid droplets in hepatocytes and activation of Kupffer cells in resveratrol-treated senescence-accelerated mice. Histochem. Cell Biol..

[bib0170] Si H., Liu D. (2014). Dietary antiaging phytochemicals and mechanisms associated with prolonged survival. J. Nutr. Biochem..

[bib0175] Takahashi S., Tamai M., Nakajima S., Kato H., Johno H., Nakamura T., Kitamura M. (2012). Blockade of adipocyte differentiation by cordycepin. Br. J. Pharmacol..

[bib0180] Tian W., Li W., Chen Y., Yan Z., Huang X., Zhuang H., Zhong W., Chen Y., Wu W., Lin C. (2015). Phosphorylation of ULK1 by AMPK regulates translocation ofULK1 to mitochondria and mitophagy. FEBS Lett..

[bib0185] Wang X.A., Xiang S.S., Li H.F., Wu X.S., Li M.L., Shu Y.J., Zhang F., Cao Y., Ye Y.Y., Bao R.F. (2014). Cordycepin induces S phase arrest and apoptosis in human gallbladder cancer cells. Molecules.

[bib0190] Wang F., Yin P., Lu Y., Zhou Z., Jiang C., Liu Y., Yu X. (2015). Cordycepin prevents oxidative stress-induced inhibition of osteogenesis. Oncotarget.

[bib0195] Wang Z.G., Li H., Huang Y., Li R., Wang X.F., Yu L.X., Guang X.Q., Li L., Zhang H.Y., Zhao Y.Z. (2017). Nerve growth factor-induced Akt/mTOR activation protects the ischemic heart via restoring autophagic flux and attenuating ubiquitinated protein accumulation. Oncotarget.

[bib0200] Worou M.E., Liao T.D., D'Ambrosio M., Nakagawa P., Janic B., Peterson E.L., Rhaleb N.E., Carretero O.A. (2015). Renal protective effect of N-acetyl-seryl-aspartyl-lysyl-proline in dahl salt-sensitive rats. Hypertension.

[bib0205] Xu Y., Liu C., Chen S., Ye Y., Guo M., Ren Q., Liu L., Zhang H., Xu C., Zhou Q. (2014). Activation of AMPK and inactivation of Akt result in suppression of mTOR-mediated S6K1 and 4E-BP1 pathways leading to neuronal cell death in invitro models of Parkinson's disease. Cell Signal..

[bib0210] Xu J., Huang Y., Chen X.X., Zheng S.C., Chen P., Mo M.H. (2016). The mechanisms of pharmacological activities of *Ophiocordyceps sinensis* fungi. Phytother. Res..

[bib0215] Yang C.H., Kao Y.H., Huang K.S., Wang C.Y., Lin L.W. (2012). *Cordyceps militaris* and mycelial fermentation induced apoptosis and autophagy of human glioblastomacells. Cell Death Dis..

[bib0220] Yang L., Song T., Chen L., Kabra N., Zheng H., Koomen J., Seto E., Chen J. (2013). Regulation of SirT1-nucleomethylin binding by rRNA coordinates ribosomebiogenesis with nutrient availability. Mol. Cell Biol..

[bib0225] Yu S.H., Dubey N.K., Li W.S., Liu M.C., Chiang H.S., Leu S.J., Shieh Y.H., Tsai F.C., Deng W.P. (2016). *Cordyceps militaris* treatment preserves renal function in type 2diabetic nephropathy mice. PLoS One.

[bib0230] Zhang C., Li C., Chen S., Li Z., Ma L., Jia X., Wang K., Bao J., Liang Y., Chen M. (2017). Hormetic effect of panaxatriol saponins confers neuroprotection in PC12 cells and zebrafish through PI3 K/AKT/mTOR and AMPK/SIRT1/FOXO3pathways. Sci. Rep..

[bib0235] Zhao B., Qiang L., Joseph J., Kalyanaraman B., Viollet B., He Y.Y. (2016). Mitochondrial dysfunction activates the AMPK signaling and autophagy to promote cell survival. Genes Dis..

[bib0240] Zhou X., Yao Y. (2013). Unexpected nephrotoxicity in male ablactated rats inducedby *Cordyceps militaris*: The Involvement of oxidative changes. Evid. Based Complement Alternat. Med..

[bib0245] Zou Y., Liu Y., Ruan M., Feng X., Wang J., Chu Z., Zhang Z. (2015). *Cordycepssinensis* oral liquid prolongs the lifespan of the fruit fly *Drosophila melanogaster*, by inhibiting oxidative stress. Int. J. Mol. Med..

