# Supplementary figures and images for: Cordyceps militaris improves the survival of Dahl salt-sensitive hypertensive rats possibly via influences of mitochondria and autophagy functions
Source: Heliyon. 2017 Nov 24;3(11):e00462. doi: 10.1016/j.heliyon.2017.e00462 (PMC5727564; doi:10.1016/j.heliyon.2017.e00462)

# Brain

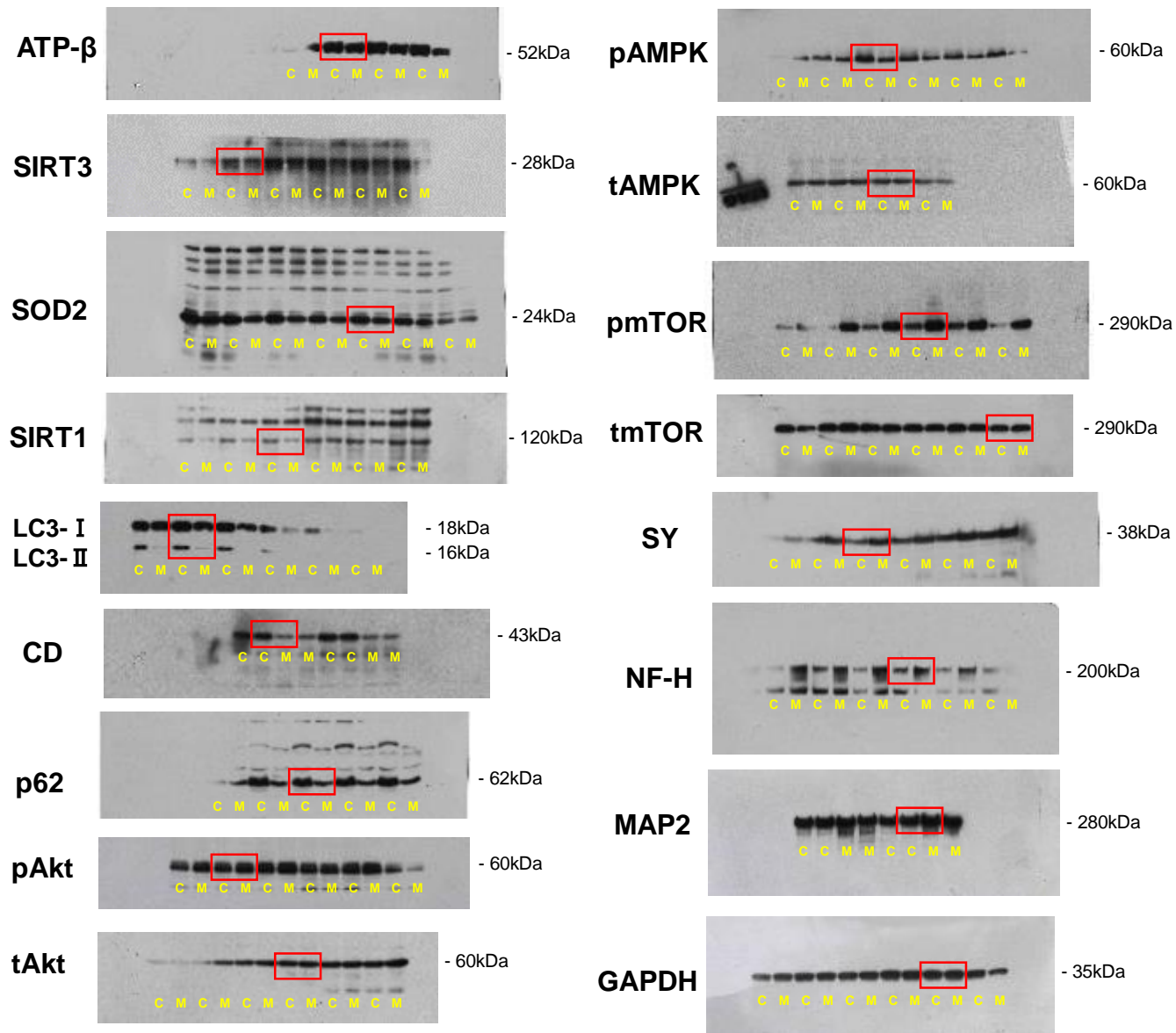

**Fig. S8.1**

Supplement: Fig. S8.1 — Brain. Full scans of original blots for data shown in Fig. 8. Panels corresponding to the images in the paper are indicated. CD, cathepsin D; SY, synaptophysin; C, Control rats; M, Cordyceps militaris (CM)-treated rats. [file mmc1.pdf]

# Spinal cord

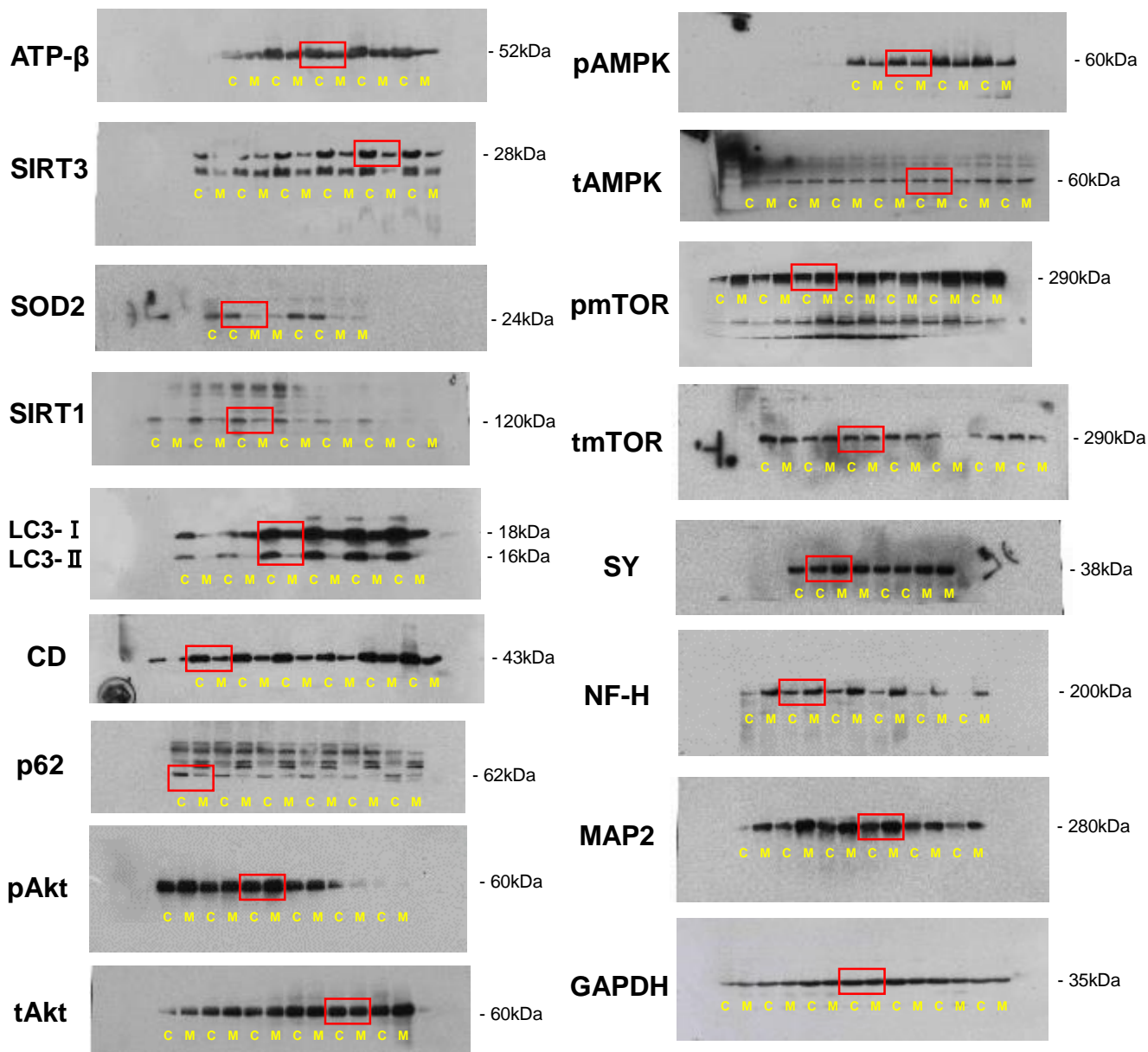

Fig. S8.2

Supplement: Fig. S8.2 — Spinal cord. Full scans of original blots for data shown in Fig. 8. Panels corresponding to the images in the paper are indicated. CD, cathepsin D; SY, synaptophysin; C, Control rats; M, Cordyceps militaris (CM)-treated rats. [file mmc2.pdf]

# Heart

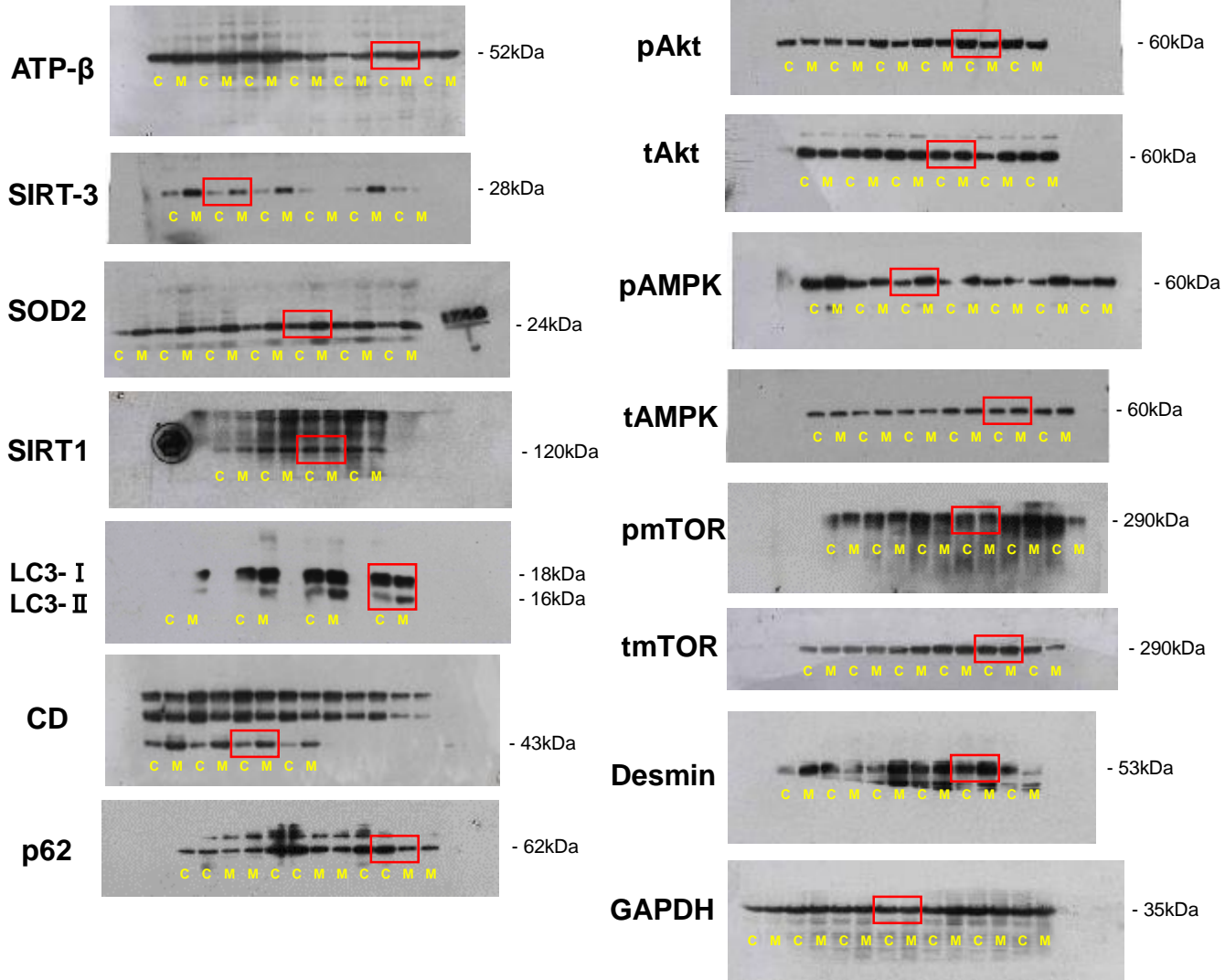

Fig. S9.1

Supplement: Fig. S9.1 — Heart. Full scans of original blots for data shown in Fig. 9. Panels corresponding to the images in the paper are indicated. CD, cathepsin D; C, Control rats; M, Cordyceps militaris (CM)-treated rats. [file mmc3.pdf]

# Kidney

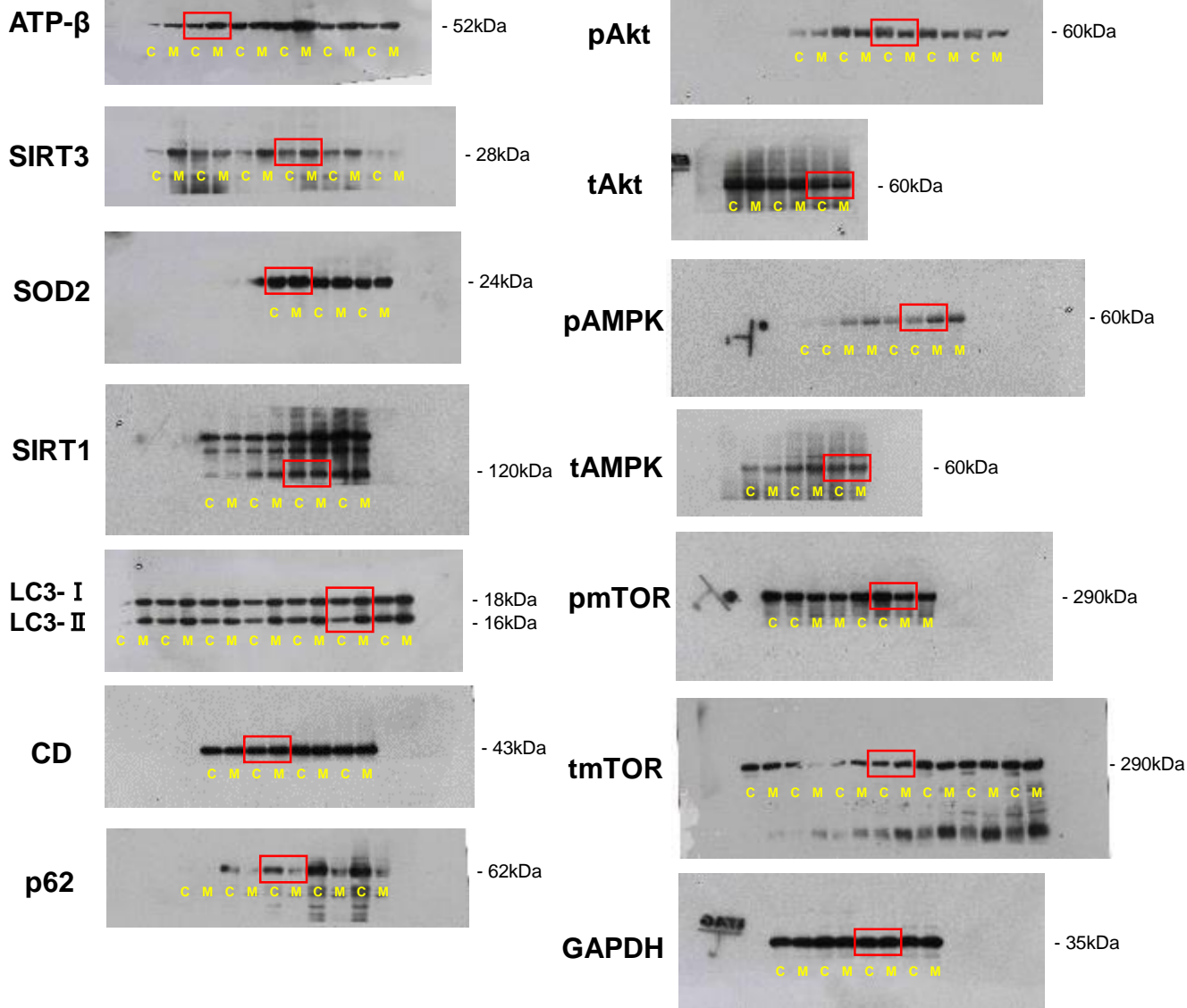

**Fig. S9.2**

Supplement: Fig. S9.2 — Kidney. Full scans of original blots for data shown in Fig. 9. Panels corresponding to the images in the paper are indicated. CD, cathepsin D; C, Control rats; M, Cordyceps militaris (CM)-treated rats. [file mmc4.pdf]

# Liver

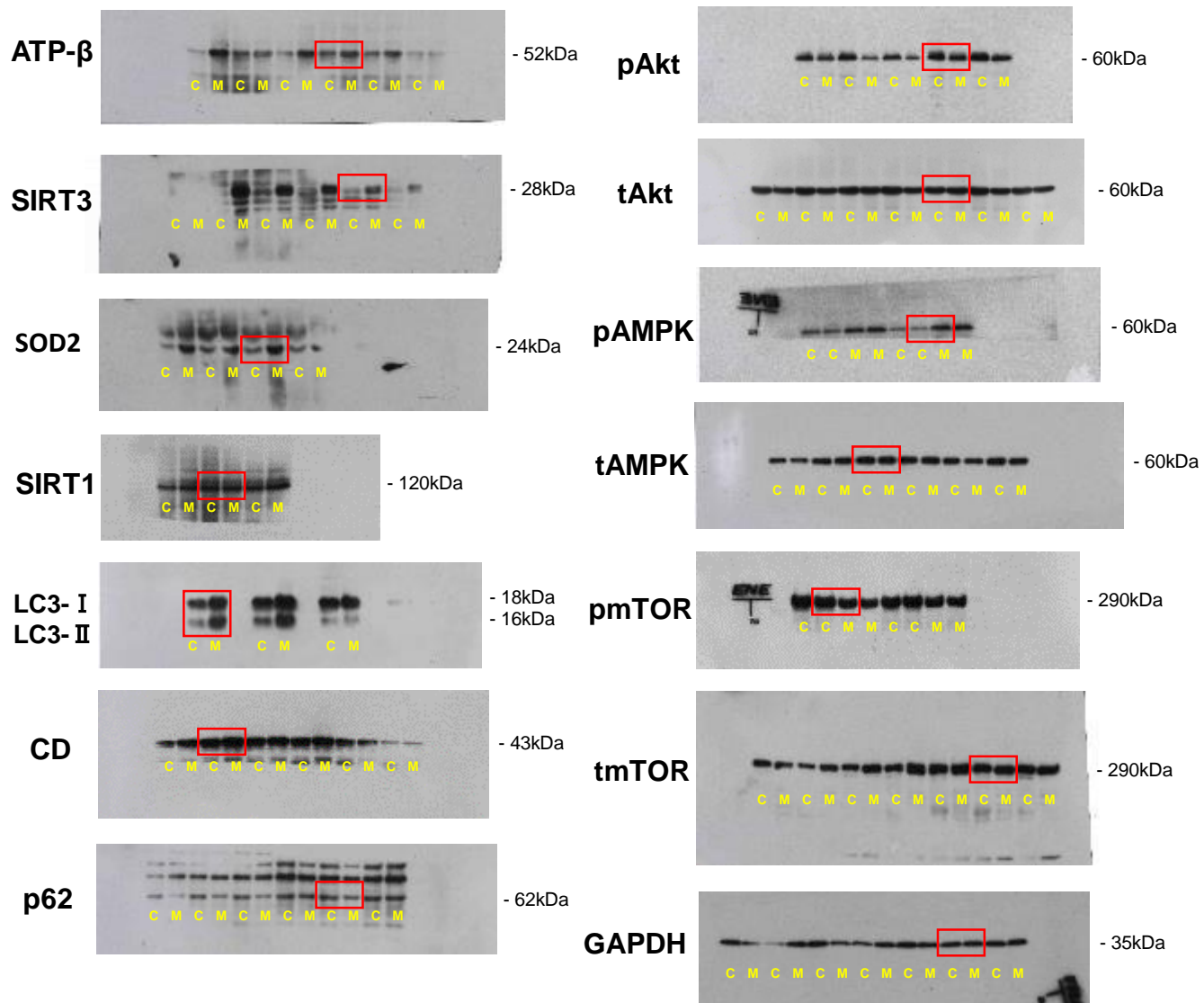

**Fig. S9.3**

Supplement: Fig. S9.3 — Liver. Full scans of original blots for data shown in Fig. 9. Panels corresponding to the images in the paper are indicated. CD, cathepsin D; C, Control rats; M, Cordyceps militaris (CM)-treated rats. [file mmc5.pdf]
